# Torix group *Rickettsia* are widespread in *Culicoides* biting midges (Diptera: Ceratopogonidae), reach high frequency and carry unique genomic features

**DOI:** 10.1111/1462-2920.13887

**Published:** 2017-09-18

**Authors:** Jack Pilgrim, Mats Ander, Claire Garros, Matthew Baylis, Gregory D. D. Hurst, Stefanos Siozios

**Affiliations:** ^1^ Institute of Infection and Global Health, Faculty of Health and Life Sciences University of Liverpool Liverpool UK; ^2^ Department of Microbiology National Veterinary Institute Uppsala Sweden; ^3^ CIRAD, UMR ASTRE Montpellier 34398 France; ^4^ CIRAD, UMR ASTRE Sainte‐Clotilde La Réunion 97490 France; ^5^ Health Protection Research Unit in Emerging and Zoonotic Infections Liverpool L69 3GL UK; ^6^ Institute of Integrative Biology, Faculty of Health and Life Sciences University of Liverpool Liverpool UK; ^7^ Present address: GE Healthcare Bio‐sciences, Björkgatan 30 Uppsala 75184 Sweden

## Abstract

There is increasing interest in the heritable bacteria of invertebrate vectors of disease as they present novel targets for control initiatives. Previous studies on biting midges (*Culicoides* spp*.)*, known to transmit several RNA viruses of veterinary importance, have revealed infections with the endosymbiotic bacteria, *Wolbachia* and *Cardinium*. However, rickettsial symbionts in these vectors are underexplored. Here, we present the genome of a previously uncharacterized *Rickettsia* endosymbiont from *Culicoides newsteadi* (RiCNE). This genome presents unique features potentially associated with host invasion and adaptation, including genes for the complete non‐oxidative phase of the pentose phosphate pathway, and others predicted to mediate lipopolysaccharides and cell wall modification. Screening of 414 *Culicoides* individuals from 29 Palearctic or Afrotropical species revealed that *Rickettsia* represent a widespread but previously overlooked association, reaching high frequencies in midge populations and present in 38% of the species tested. Sequence typing clusters the *Rickettsia* within the Torix group of the genus, a group known to infect several aquatic and hematophagous taxa. FISH analysis indicated the presence of *Rickettsia* bacteria in ovary tissue, indicating their maternal inheritance. Given the importance of biting midges as vectors, a key area of future research is to establish the impact of this endosymbiont on vector competence.

## Introduction

Heritable bacteria represent an important component of the biology of many arthropods. Carried by over half of all species (Weinert *et al*., 2015), many vertically transmitted microbes contribute to host function. This contribution is most commonly through specific services, such as nutrient provisioning or protection (Oliver *et al*., 2003; Douglas, 2009; Jaenike *et al*., 2010). Conversely, their maternal‐inheritance has led symbionts to favour production of daughters by their host, leading to the evolution of systems biasing offspring sex ratio towards females (reproductive parasitisms) (Hurst and Frost, 2015). The strength of symbiont impact on individual biology, combined with the high frequency with which arthropod species are infected with symbionts, has led to intense study. This study has the complementary motivations of understanding the dynamics and ecological impact of symbionts (Ferrari and Vavre, 2011) and applying this knowledge to modify the biological properties of target species (Iturbe‐Ormaetxe *et al*., 2011).

Particular attention has been focused on symbiont/host interactions in vector species. Through the induction of cytoplasmic incompatibility, the endosymbiont *Wolbachia* prevents the formation of viable progeny between infected males and uninfected females in various dipterans including *Drosophila* spp. and *Aedes* spp. (Werren *et al*., 2008). With respect to the latter, not only can this incompatibility lead to vector population suppression but, through unknown mechanisms, a strong RNA virus resistance phenotype (Moreira *et al*., 2009; Bian *et al*., 2010; Blagrove *et al*., 2012; Van den Hurk *et al*., 2012). Furthermore, experimental evidences show that both *Wolbachia* and another proteobacteria, *Wigglesworthia*, can act as obligate (required) symbionts, provisioning blood sucking vector hosts with B vitamins that are lacking in a blood‐diet (reviewed in Rio *et al*., 2016). This provisioning has evolved independently in bed bugs (*Cimex lectularius*) (Nikoh *et al*., 2014) and tsetse flies (*Glossina* sp.) (Akman *et al*., 2002; Snyder *et al*., 2010; Rio *et al*., 2012). Additional genomic surveys suggest that other proteobacterial symbionts including *Coxiella* are involved in metabolic homeostasis (Zhong *et al*., 2007; Manzano‐Marin *et al*., 2015; Smith *et al*., 2015). As such, these symbioses can have profound effects on the biology, ecology and evolutionary dynamics of vector–pathogen interactions.

*Rickettsia* (class: Alphaproteobacteria; order: Rickettsiales) symbionts are obligate intracellular bacteria most notable for containing species pathogenic to vertebrates, such as *Rickettsia prowazekii*, the causative agent of louse‐borne Typhus fever, *Rickettsia rickettsii* (Rocky Mountain spotted fever) and *Rickettsia conorii* (Boutonneuse or Mediterranean spotted fever). Despite this, vertebrate disease‐causing *Rickettsia* are atypical of the genus as a whole (Perlman *et al*., 2006; Weinert *et al*., 2009a) and many *Rickettsia* are maintained without infectious transfer. Members are known to induce a variety of reproductive manipulations, including male killing in ladybird beetles (*Adalia bipunctata*) (Werren *et al*., 1994; Hurst *et al*., 1999; Majerus *et al*., 1999) and parthenogenesis induction in parasitoid wasps (*Pnigalio soemius*; *Neochrysocharis formosa*) (Hagimori *et al*., 2006; Giorgini *et al*., 2010). *Rickettsia* symbiont infection can also be protective, enhancing resistance of aphids (*Acyrthosiphon pisum*) to fungal attack and whiteflies (*Bemisia tabaci*) to bacterial challenge (Łukasik *et al*., 2013; Hendry *et al*., 2014). Of significance to the study of vectors, *Rickettsia* are also known to increase the competence of *Bemisia* whiteflies for the transmission of tomato leaf curl virus (Kliot *et al*., 2014). Members of the genus can also be insect‐vectored plant pathogens in their own right, for example, underlying papaya bunchy top disease (Luis‐Pantoja *et al*., 2015). As such, symbiosis with *Rickettsia* is biologically important at the individual and population levels and both as vectored disease agents in themselves and as a symbiont facilitating the spread of other diseases.

In this work, we uncovered and examined a symbiotic association between *Rickettsia* and *Culicoides* biting midges which has been previously overlooked. Worldwide, biting midges of the genus *Culicoides* (Diptera: Ceratopogonidae) are known to transmit more than 50 arboviruses as well as some nematode and protozoan parasites. Midge‐vectored pathogens that threaten livestock and wildlife include bluetongue virus (BTV), Schmallenberg virus, African horse sickness virus, epizootic hemorrhagic disease virus, equine encephalosis virus and Akabane virus (Mellor *et al*., 2000). In South America, *Culicoides* midges spread Oropouche virus to humans. Previous studies of *Culicoides* symbionts have screened extensively for *Cardinium* and *Wolbachia* infections (Nakamura *et al*., 2009; Morag *et al*., 2012; Lewis *et al*., 2014; Mee *et al*., 2015) but failed to report presence of *Rickettsia*. However, a 16S metagenomic screening project in *Culicoides sonorensis* gut samples revealed amplicons allied to *Rickettsia* (Campbell *et al*., 2004), albeit with no phylogenetic or population‐based information. Complementary to this, when we performed a shallow whole‐genome sequencing of the *Cardinium*‐uninfected midge *Culicoides newsteadii* N5, we recovered a near complete genome of an uncharacterized, divergent *Rickettsia* species related to the Torix (also known as Limoniae) group of *Rickettsia*.

In this study, we first report on the genomic properties of the *Rickettsia* endosymbiont of *C. newsteadi* (RiCNE), which represents the first *Rickettsia* genome from the Torix group. We then examine the distribution and prevalence of *Rickettsia* in a wide‐range of *Culicoides* species from both Palearctic and Afrotropical regions and resolve the relationship of the *Culicoides Rickettsia* based on five gene sequences. We conclude that *Rickettsia* infection is common in *Culicoides* and raise the hypothesis that Torix group *Rickettsia* may be a dominant taxon in invertebrates with aquatic stages. Our genome data provide no support for a symbiont role in vitamin homeostasis but reveal unique features potentially related to the ecological attributes of this *Rickettsia* group.

## Results

### Serendipitous discovery of a *Rickettsia* symbiont during the shallow sequence of its *Culicoides* midge host

*Culicoides newsteadi* N5 is morphologically and genetically similar to *C. punctatus*, which has been previously reported to be infected with *Cardinium* symbiotic bacteria (Lewis *et al*., 2014). During a shallow illumina whole genome sequencing of *C. newsteadi* N5, we identified the presence of several contigs with homology to *Rickettsia* bacteria (Supporting Information Fig. S1).

### General features and genetic repertoire of the RiCNE draft genome

**Figure 1:**
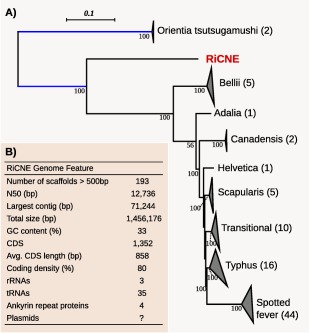
The *Rickettsia* endosymbiont of *C. newsteadi* N5 (RiCNE). A. Phylogenomic placement of RiCNE was inferred using maximum likelihood (RaxML, model: Lag + G + I) from the concatenated protein alignments of 189 single copy ortholog genes. Support values are based on 100 rapid bootstrap replicates. Major *Rickettsia* groups have been collapsed for visualisation purposes and names are according to (Murray *et al*., 2016). Numbers in parenthesis represent the number of genomes used for the analysis (Table S2). The blue branches have been reduced 50% for visualisation purposes. The full phylogenetic tree is shown in Supporting Information Fig. S4. B. RiCNE draft genome features. [Color figure can be viewed at wileyonlinelibrary.com]

The final assembly of the RiCNE draft genome consists of 193 scaffolds > 500 bp (N50 = 12.7 kb, largest scaffold = 71.2 kb) comprising a total size of 1,456,176 bp with an average GC content of 33% and an average depth of coverage 76× (Fig. 1B). Genome annotation identified 1352 protein coding sequences (CDSs) with an average length of 858 bp, a full set of rRNA genes (one each of 16S, 5S and 23S) and 35 tRNA genes accounting for a coding density of circa 80%. The proportion of missing BUSCO marker genes in RiCNE draft assembly fell well within the range of the previously completely sequenced *Rickettsia* genomes [BUSCO score = C: 93.2% (S: 93.2%, D: 0%), F: 0%, M: 6.8%, *n*: 148] (Supporting Information Fig. S2). These results suggest that the RiCNE draft assembly represents a nearly complete genome. From the 1352 predicted CDSs, 962 (∼ 71%) CDSs were annotated with putative functions, while 390 (∼ 29%) CDSs were annotated as hypothetical proteins. Additional searches for Pfam domains revealed that 122 of the hypothetical proteins had putative functional domains (Supporting Information Table S1).

### Phylogeny

**Figure 2:**
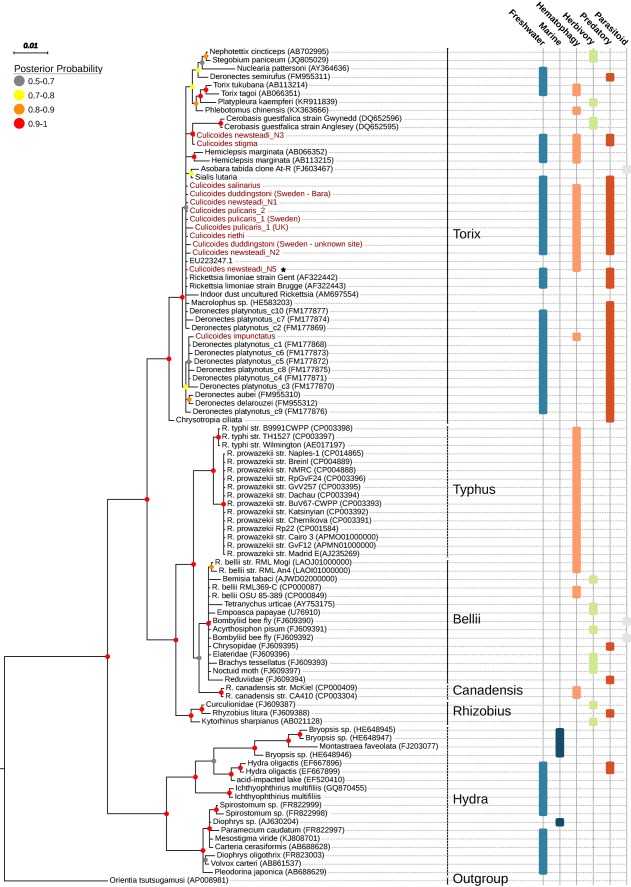
Phylogenetic placement of the *Rickettsia* symbionts of *Culicoides* midges based on the 16S rRNA gene. Previously characterized *Rickettsia* groups including the basal group of Hydra are also presented. Host names are used in the absence of official *Rickettsia* species name. Tree topology and posterior probabilities (shown as color‐coded circles on each node) were inferred using Bayesian analysis in MrBayes under GTR + G + I (for details see the ‘Experimental procedures’ section). Sequence accession numbers are shown in brackets. A black star depicts the midges *Rickettsia*, which draft genome is presented in this study. The habitats and lifestyles of the host (or specific life stages of the host) are given to the right of the phylogeny. [Color figure can be viewed at wileyonlinelibrary.com]

The phylogenetic relationships of RiCNE relative to other Rickettsiaceae were initially estimated from a set of 189 single copy panorthologs identified among 84 complete or draft *Rickettsia* genomes and its sister genus *Orientia* (Supporting Information Table S2). Maximum‐likelihood phylogeny placed RiCNE as a sister lineage of all other *Rickettsia* with strong support (bootstrap support = 100%) (Fig. 1A). Additionally, we performed a phylogenetic analysis using the conserved 16S rRNA which allowed us to include representative sequences from the Hydra and Torix groups of *Rickettsia* (Weinert *et al*., 2009a). Our analyses clearly positioned RiCNE sequence within the Torix group (Fig. 2) previously identified in leeches (Kikuchi *et al*., 2002), amoebae (Dyková *et al*., 2003) and several arthropod orders including Araneae, Diptera, Coleoptera, Psocoptera, Hemiptera and Hymenoptera (Goodacre *et al*., 2006; Perotti *et al*., 2006; Reeves *et al*., 2008; Küchler *et al*., 2009; Zouache *et al*., 2009; Machtelinckx *et al*., 2012; Weinert *et al*., 2015). The RiCNE genome represents the first to be sequenced from this group.

### Genome content

We compared the content of the RiCNE genome with other Rickettsiaceae (Supporting Information Table S2) to identify unique features potentially related to the biology of RiCNE and the Torix group *Rickettsia*. Overall, RiCNE presents typical features and genetic repertoire of a *Rickettsia* genome including the presence of a P‐like type IV secretion system (P‐T4SS) which is highly conserved among Rickettsiales (Supporting Information Fig. S3 and Table S3). The *vir* genes on the RiCNE genome are arranged into three major clusters (scaffold 1: *vir*B3, *vir*B4 and *vir*B6; scaffold 4: *vir*B8‐B11 and *vir*D4; scaffold 10: two in tandem paralogs of the *vir*B2 gene and a *vir*B4 paralog) with additional paralogs of the *vir*B8 and *vir*B9 on scaffold 47. This scattered arrangement of the *vir* genes is typical of *Rickettsia* genomes (Gillespie *et al*., 2009). Additionally, the RiCNE genome encodes a *tra* conjugative DNA‐transfer element, which has been previously reported in several *Rickettsia* genomes (Ogata *et al*., 2006; Weinert *et al*., 2009b). RiCNE *tra* cluster is split into two scaffolds (scaffolds 5 and 34). The first unit contains the ‘F‐like’ T4SS (*tra*) genes including *tra*E, *tra*K, *tra*B, *tra*C, *tra*W, *tra*U, *trb*C, *tra*N, *tra*F, *tra*H and *tra*G_N (Supporting Information Fig. S3 and Table S3). The second unit contains the ‘Ti‐like’ genes *tra*A_Ti_ and *tra*D_Ti_ previously identified in the Ti plasmid of *Agrobacterium tumefaciens* (Wood *et al*., 2001). Although we could not identify a *tra*V homolog (a core glycoprotein, component of the pilus assembly structure), a hypothetical protein encoded by a gene located between the *tra*B and *tra*C homologs presented low similarities with TraV homologs from the *Rickettsia* endosymbiont of *Ixodes scapularis* and may represent a functional equivalent. Notably, the two scaffolds containing the conjugation genes were consistently represented at 2–3 times higher than average coverage (Supporting Information Fig. S3). However, this does not exceed the even higher coverage associated with repetitive loci such as insertion elements. This suggests that the conjugation system genes are likely encoded as multiple copies on the chromosome, as previously reported for other *Rickettsia* and *Orientia* (Cho *et al*., 2007; Gillespie *et al*., 2012). However, the presence of low‐copy‐number plasmids cannot be ruled out.

A shared feature among *Rickettsia* is the presence of several gene families potentially involved in environmental adaptation. These include multiple paralogous genes encoding the bifunctional (p)ppGpp synthase/hydrolase SpoT/RelA, a key component of the bacterial stringent response, several genes related to toxin‐antitoxin systems (see below) as well as genes encoding multidrug/efflux transporters. In the RiCNE genome, we identified 18 CDSs with homology to *spo*T paralogs shared with other *Rickettsia* genomes. Five of them were found at the ends of the scaffolds and may represent incomplete fragments, while another two truncated CDSs occurred in tandem and may represent a pseudogene.

A total of 187 of the 1352 predicted CDSs (∼ 14%) were unique to the RiCNE genome. Of these 187 CDSs, 43 CDSs were predicted to form hypothetical proteins of less than 70 amino acids and may therefore represent annotation artefacts or pseudogenised gene fragments. Forty of the remaining 144 RiCNE‐specific CDSs could be ascribed a putative function, either by significant matches in the NR database or by predicted Pfam domains (Supporting Information Table S4). Amongst these were genes putatively associated with host invasion and host–microbe interactions. These include a homolog of a putative exopolysaccharide synthesis (*exo*D) gene (RiCNE_02810), two paralogs of a putative lipid A 3‐*O*‐deacylase (*pag*L) gene (RiCNE_02710, RiCNE_13110), as well as a gene coding for a carbonic anhydrase (RiCNE_13200) and a gene coding for a leucine‐rich repeat protein (RiCNE_13500) (Supporting Information Table S5). Moreover, we identified four genes encoding cell wall biogenesis and modification proteins including UDP‐galactopyranose mutase (RiCNE_08860), *N*‐acetylmuramoyl‐l‐alanine amidase (RiCNE_08880), a putative Glycosyl transferases (RiCNE_08940) and a putative d‐alanyl‐d‐alanine carboxypeptidase (RiCNE_06020). Multiple genes coding for toxin–antitoxin systems (13 toxins and 9 antitoxins) were also detected. Of these, two CDSs encoding for a toxin (RiCNE_11100) and an antitoxin (RiCNE_07550) were specific to RiCNE. Finally, among the 14 multidrug/efflux transporters identified, two (RiCNE_09880 and RiCNE_13240) are specific to RiCNE.

### The metabolic and biosynthetic potential of RiCNE

**Figure 3:**
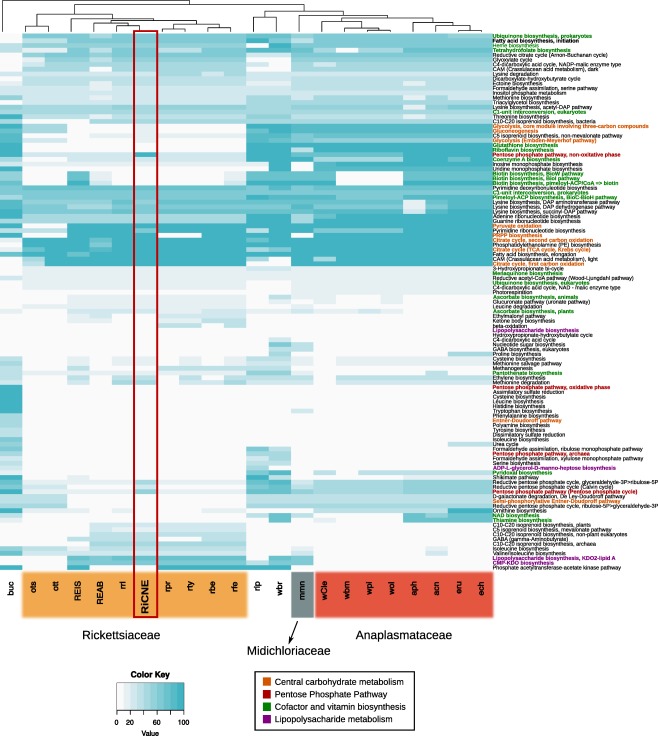
Assessment of metabolic potential of RiCNE genome (highlighted) and comparison to other members of the order Rickettsialles as well as representative known nutritional mutualists (primary symbionts) *Buchnera*, *Riesia* and *Wigglesworthia*. Color gradient represents the module completion ratio (MCR) values calculated by MAPLE‐2.1.0. buc: *Buchnera aphidicola* APS, ots: *Orientia tsutsugamushi* Boryong, ott: *Orientia tsutsugamushi* Ikeda, REIS: *Rickettsia* endosymbiont of *Ixodes scapularis*, REAB: *Rickettsia* endosymbiont of *Adalia bipunctata*, rri: *Rickettsia rickettsii* Sheila Smith, RiCNE: *Rickettsia* endosymbiont of *Culicoides newsteadi*, rpr: *Rickettsia prowazekii* Madrid E, rty: *Rickettsia typhi* Wilmington, rbe: *Rickettsia bellii* RML369‐C, rfe: *Rickettsia felis*, rip: *Candidatus* Riesia pediculicola, wbr: *Wigglesworthia glossinidia* brevipalpis, mmm: *Candidatus* Midichloria mitochondrii, wcl: *Wolbachia w*Cle, wbm: *Wolbachia w*Bm, wpi: *Wolbachia w*Pip, wol: *Wolbachia w*Mel, aph: *Anaplasma phagocytophilum* HZ, acn: *Anaplasma centrale*, eru: *Ehrlichia ruminantium* Welgevonden and ech: *Ehrlichia chaffeensis* Arkansas. [Color figure can be viewed at wileyonlinelibrary.com]

Overall, the metabolic capacities of the RiCNE genome are similar to other *Rickettsia* genomes. Like other *Rickettsia*, it is missing several central aspects of metabolism such as the glycolysis and gluconeogenesis pathways (Fig. 3). Likewise, pathways for nucleotide and amino acid biosynthesis are absent or defective. Instead, we identified genes encoding for putative transporters including five ATP/ADP translocase homologs, two amino acid permeases and several putative transporters belonging to major facilitator super‐family (MFS), suggesting that RiCNE likely relies on the exploitation of host resources.

**Figure 4:**
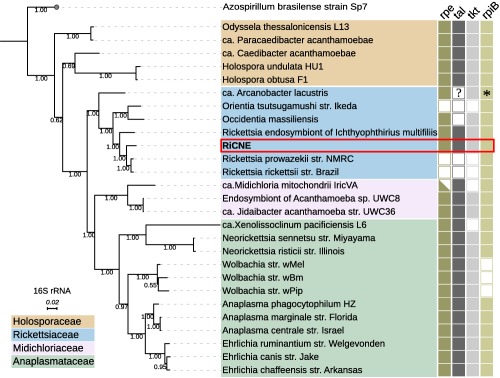
Loss of the non‐oxidative stage of the pentose phosphate pathway (PPP) among Rickettsiales. Presence and absence of the four key enzymes (rpe: ribulose‐phosphate 3‐epimerase, tla: transaldolase, tkt: transketolase and rpi: ribose 5‐phosphate isomerase) are shown across the 16S rRNA phylogeny of representative members from the Rickettsiales as filled and empty rectangles, respectively. Completeness of the pathway in RiCNE is highlighted. Half‐filled rectangle indicates a truncated rpe gene homologue. The question mark indicates missing data rather than loss of tla homolog from *ca*. Arcanobacter lacustris since its genome is incomplete (completeness = 48%; Martijn *et al*., 2015). The asterisk indicates a possible gene fusion event in ca. Arcanobacter lacustris between the rpiB gene and the upstream gene coding for a NAD(P)H: quinone oxireductase. 16S rRNA tree constructions were performed with MrBayes under GTR + G + I. A phylogenetic analysis based on the individual protein sequences is presented in Supporting Information Fig. S5. [Color figure can be viewed at wileyonlinelibrary.com]

A marked difference between RiCNE genome and all other sequenced Rickettsiaceae is that RiCNE encodes the complete set of proteins involved in the non‐oxidative phase of the pentose phosphate pathway (PPP), including transketolase, transaldolase, ribulose‐phosphate 3‐epimerase and a ribose 5‐phosphate isomerase B (RiCNE_05410, RiCNE_04320, RiCNE_00410 and RiCNE_09330, respectively) (Fig. 3). The oxidative phase is completely absent, as for other *Rickettsia*. Only one gene of the PPP (coding for the ribose 5‐phosphate isomerase B) has been detected in most other sequenced Rickettsiaceae including *Orientia tsutsugamusi*. To better understand the evolution of the non‐oxidative PPP branch in *Rickettsia*, we search the unpublished genome of the *Rickettsia* endosymbiont of *Ichthyophirius multifilis* for the presence of the same four key proteins. This rickettsial endosymbiont is affiliated to the basal Hydra group of *Rickettsia* (Weinert *et al*., 2009a,b) (Fig. 2) commonly found among diverse ciliates and recently provided with the unique genus name *Megaira* (Schrallhammer *et al*., 2013). This genome was obtained by sequencing its ciliate host (Sun *et al*., 2009) and was kindly provided by Prof. R.S. Coyne, Dr T. Doak and Dr H. Suzuki. Notably, all four proteins were encoded in this *Rickettsia* endosymbiont genome displaying moderate amino‐acid sequence similarity with the RiCNE homologs (rpe: 58.6%, tal: 58.1%, tkt: 52.2% and rpiB: 63.6%). We additionally conducted protein similarity searches against the NR database (NCBI) using the three sequences that did not have any homologs among the arthropod‐associated *Rickettsia*. The best BLAST hits for all three sequences fell within the α‐proteobacteria (RiCNE_05410 shared ∼ 50% amino acid identity with *Ehrlichia* homologs, RiCNE_04320 shared ∼ 59% identity with *Sulfitobacter* sp. EhC04 and RiCNE_00410 shared ∼ 58% identity with an uncultured α‐proteobacterium). Additional phylogenetic analyses of the individual PPP protein sequences clearly cluster the RiCNE sequences within the alpha‐proteobacteria and the Rickettsiales, and partial PPPs were detected in other members of the Rickettsiales including *Wolbachia* and *Midichloria* (Fig. 4). Finally, we also noticed that RiCNE_05410 gene contains an in‐frame insertion of a *Rickettsia* Palindromic Element (RPE) between the positions 1560 and 1666.

Inspection of predicted biosynthetic pathways for cofactors and B vitamin synthesis systems revealed no major differences from the rest of the Rickettsiaceae (Fig. 3). RiCNE features a reduced set of genes required for folate (vitamin B9) biosynthesis, with the gene for dihydrofolate synthase (*fol*C) absent. The pathways required for the biosynthesis of biotin (vitamin B7), riboflavin (vitamin B2), thiamin (vitamin B1), pyridoxine (vitamin B6), nicotinate (vitamin B3) and pantothenate (vitamin B5) are completely absent. Moreover, the cofactor biosynthetic capacity of RiCNE appears to be limited, with only partial pathways for heme and ubiquinone biosynthesis.

### Prevalence of *Rickettsia* in biting midges

Screening of field collected midge specimens revealed *Rickettsia* infections in 155 of 414 (37%) individuals and 11 of 29 (38%) *Culicoides* species sampled (Table 1 and Supporting Information Table S6). *Rickettsia*‐positive species of biting midge were recorded across *Culicoides* subgenera. Infection was identified across the subgenera Beltranmyia (1/3 species), Culicoides (7/11 species), Monoculicoides (2/2) and Oecacta (1/4 species) [as determined by Borkent (2016)]. There was no apparent host sex bias in the presence of *Rickettsia* for either *Culicoides pulicaris* haplotype 1 (UK) (Fisher's two‐tailed test; *p* = 1) or *Culicoides impunctatus* (Fisher's two‐tailed test; *p* = 0.36), the only infected species with both host sexes available to compare.

**Table 1:** *omp* conventional PCR assay results for *Rickettsia*‐positive *Culicoides* sp. under study, given by subgenus, species, location, date and sex.

				Proportion of *Rickettsia*‐positive samples (*n*) [95% confidence interval]	
Subgenus	*Culicoides* species	Location	Year of collection	Females	Males
Beltranmyia	*C. salinarius*	Unknown site, Sweden	2009	1 (2) [0.2–1]	
Culicoides	*C. impunctatus*	Torsås, Sweden	2008	0.3 (20) [0.13–0.54]	
		Bala, UK	2012	0.81 (17) [0.5–0.92]	0.5 (14) [0.27–0.73]
		Kielder, UK	2016	0.75 (16) [0.47–0.92]	0.86 (7) [0.42–0.99]
	*C. newsteadi* N1^a^, *	Site 2, Corsica	2015	0.5 (2) [0.1–0.91]	
	*C. newsteadi* N2^a^, *	Site 2, Corsica	2015	1 (2) [0.2–1]	
	*C. newsteadi* N3^b^, *	Unknown site, Sweden	2008–2010	1 (6) [0.52–1]	
	*C. newsteadi* N5^b^, *	Wirral, UK	2015	1 (13) [0.72–1]	
	*C. pulicaris* * (haplotype 1)	Canterbury, UK	2014	1 (2) [0.2–1]	
		Hereford, UK	2014	1 (1) [0.05–1]	
		Luton, UK	2014	1 (1) [0.05–1]	
		Unknown site, Sweden	2008–2010	1 (6) [0.52–1]	
		Wirral, UK	2015	1 (32) [0.87–1]	
		Wolverhampton, UK	2013	1 (11) [0.68–1]	1 (4) [0.4–1]
		Worcester, UK	2014	1 (6) [0.52–1]	
	*C. pulicaris* ^*^ (haplotype 2)	Site 2, Corsica	2015	1 (13) [0.72–1]	
Monoculicoides	*C. riethi*	Ljungbyholm, Sweden	2010	1 (1) [0.05–1]	
	*C. stigma*	Unknown site, Sweden	2008	1 (3) [0.31–1]	
Oecacta	*C. duddingstoni*	Bara, Sweden	2008	1 (4) [0.4–1]	
		Unknown site, Sweden	2008–2010	1 (3) [0.31–1]	

**a.** *Culicoides newsteadi* haplotypes are designated by Pagès *et al*. (2009).

**b.** *Culicoides newsteadi* haplotypes are designated by Ander *et al*. (2013).

*C. newsteadi and *C. pulicaris* haplotypes are defined as separate species as their *COI* barcodes have < 81% identity between each other.

*Rickettsia* was found at fixation in all individuals in 16 of the 20 positive populations screened, being at low or intermediate prevalence in the remaining 4 (1 *C. newsteadi* N1 population and 3 *C. impunctatus* populations). Where multiple samples of particular species were tested, there was no significant difference in the fraction infected (*C. impunctatus* populations from Bala vs. Kielder in the UK, N1 = 31, N2 = 23, Fisher's two‐tailed test; *p* = 0.37). Mitochondrial DNA barcoding of infected (KY765353) and uninfected (KY765354) individuals of *C. impunctatus* confirmed these individuals shared a barcode, consistent with infection showing within‐species polymorphism.

### Rickettsia diversity in *Culicoides*

The level of 16S rRNA divergence within the *Culicoides Rickettsia* was low (0.9% segregating sites, Pi = 0.002) (Supporting Information Table S7), such that the strains would all be considered as belonging to a single species in classic bacteriological nomenclature (Stackebrandt and Goebel, 1994). To resolve patterns of relatedness more fully, we obtained the sequence of three further housekeeping loci as well as the *omp* gene, for each of the specimens. Housekeeping gene PCR amplification was successful for 13 typings; The *C. pulicaris* strain (I) from the UK failed to amplify with the COX primers after more than one attempt. An exclusive allele was designated to this locus, because non‐amplification implies the genotype of this strain is unique at the priming site, as failure to amplify occurred on a background of successful amplification for other loci in these specimens. The number of alleles per locus ranged from 6 to 10, with a total of 11 unique allelic profiles found (Supporting Information Table S8). All gene sequences, including the non‐housekeeping gene *omp*, maintained an intact coding frame, consistent with their presence in a symbiont genome, rather than a nuclear insertion of a *Rickettsia* gene. The most polymorphic housekeeping locus was a*tpA*, with 9.9% variable sites and the highest level of nucleotide diversity per site (Pi = 0.046) (Supporting Information Table S7). This gene exhibited evidence of intragenic recombination suggested by atypical pairwise divergence in closely related isolates (Supporting Information Table S9), as well as detection by RDPv4 (Martin *et al*., 2015) (*p* < 0.001, determined by MaxChi) (Supporting Information Table S7). All genes showed average *K*_a_/*K*_s_ of less than 1 (Supporting Information Table S7), indicating that the genes were subject to purifying selection, conforming to the general requirements for reliable indicators of genetic relatedness between bacterial isolates. Predictably, as an antigenic protein with less intense purifying selection and potential episodes of positive selection, *omp* had a greater average *K*_a_/*K*_s_ than the other loci, although no signs of positive selection were observed at the gene‐level.

Whilst there was evidence that the strains found within *Culicoides* were closely related, it is not clear if they are monophyletic. Some loci demonstrated 100% sequence identity with *Rickettsia* strains from other taxa. These included the partial *gltA* sequences of *C. impunctatus*, which was identical to the *Rickettsia* symbionts of the beetle *Deronectes platynotus* (Dytiscidae; FM177878) (Küchler *et al*., 2009), the Dipteran fly *Chrysotimus flaviventris* (Dolichopodidae; JQ925578) (Martin *et al*., 2013) and the spider *Pityohyphantes phrygianus* (Linyphiidae; DQ 231491) (Goodacre *et al*., 2006), and the partial 16S sequences of clonal complex 2 strains (*C. duddingstoni* (Bara, Sweden), *C. pulicaris* haplotype 1 (Sweden), *C. newsteadi* N1, *C. pulicaris* (haplotype 2), which were identical to the 16S sequence of the *Rickettsia* in the cranefly *Limonia chorea* (Limoniidae; AF322443). Furthermore, a coxA 995 bp region of the Hemipteran bug *Macrolophus* sp. *Rickettsia* 1 (Miridae; HE583223) (Machtelinckx *et al*., 2012) was > 99% similar to all *Culicoides*’ strains except for *C. impunctatus* and *C. salinarius*. Moreover, enforcing the monophyly of *Culicoides Rickettsia* on the 16S phylogeny (Fig. 2) did not result in a significantly worse tree (SH‐test, *p* > 0.05). Similar results were obtained when a phylogenetic analysis was conducted using the available *Rickettsia glt*A sequences (data not shown). Thus, it is unclear (largely due to lack of multi locus data from other taxa) whether the *Culicoides Rickettsia* represents a monophyletic group.

**Figure 5:**
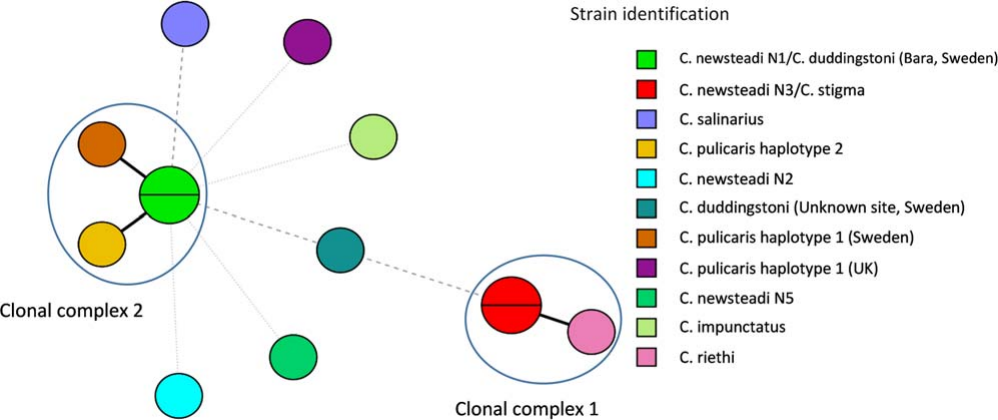
Minimum spanning tree using Unweighted Pair Group Method with Arithmetic Mean (UPGMA) cluster analysis of isolates. Allelic profiles that are shared by more than one host species are designated as central strains (CSs). Strains differing at one locus (SLVs) are connected by a solid line, strains sharing one or more loci are connected by a dashed line and unique strains sharing no allele identity are connected by a faded line. [Color figure can be viewed at wileyonlinelibrary.com]

We next examined the relationship of the *Rickettsia* strains from different host species using allelic profiles across loci (Fig. 5). Most allelic profiles obtained from different host populations (11/13) were unique. Furthermore, of these 11 unique allelic profiles, 4 (H, I, J and K) allelic profiles shared no alleles with other strains. Allelic profiles that were shared by more than one host species were designated as central strains (CSs), whereas isolates that varied at one locus to these CSs were termed single locus variants (SLVs). Together the CSs and SLVs form clonal complexes, as they are presumed to be closely related. Two clonal complexes were identified in this study (Fig. 5); the central strain A from *C. stigma* and *C. newsteadi* N3 formed clonal complex 1 with the SLV strain from *C. riethi* (B), whereas the central strain C from *C. newsteadi* N1 and *C. duddingstoni* (Bara, Sweden) formed clonal complex 2 with the SLV strains from *C. pulicaris* haplotype 1 (Sweden) (D) and *C. pulicaris* haplotype 2 (E).

### Visualisation of *Rickettsia* in *C. impunctatus*’ ovaries

**Figure 6:**
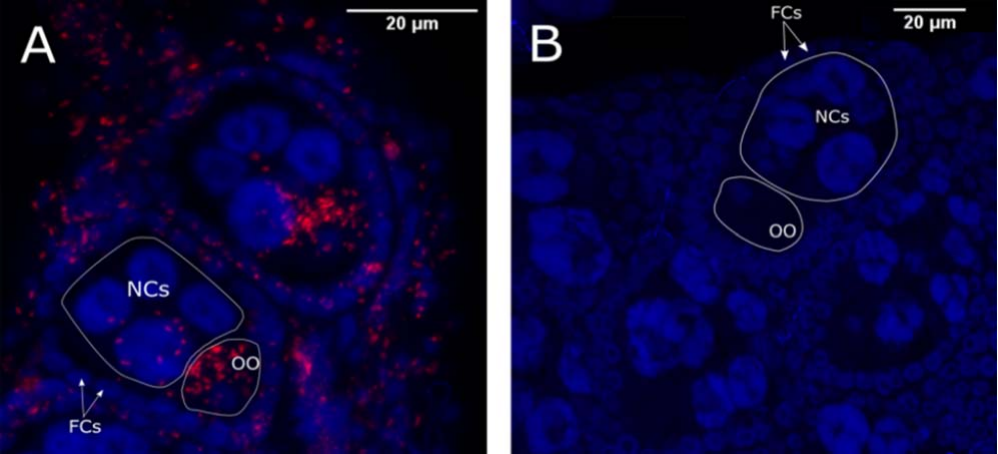
*Rickettsia* localization in midge ovaries via FISH. The combined z‐stack optical sections of infected *C. impunctatus* (A) and *Rickettsia* free *C. nubeculosus* (B) ovarioles stained with DAPI (blue) and an ATTO633‐labeled Rickettsia‐specific probe (red). FCs: follicle cells, NCs: nurse cells and OO: oocyte. [Color figure can be viewed at wileyonlinelibrary.com]

Fluorescent *in situ* hybridization (FISH) of *C. impunctatus*’ dissected ovaries, using a *Rickettsia*‐specific probe, showed strong positive signals within the ovarioles (Fig. 6A). The strongest signal was localized inside the developing oocytes. In addition, hybridization signals were detected inside nurse and follicle cells. No signal was detected in the *Rickettsia*‐uninfected controls used (Fig. 6B), suggesting the specificity of the detection.

## Discussion

In this study, we serendipitously recovered the genome of a *Rickettsia* bacterium (RiCNE) from the WGS sequencing of *C. newsteadi* N5, the 16S rRNA sequence of which paralleled a *Rickettsia* identified in a screen of the midgut microbiota of *C. sonorensis* (Campbell *et al*., 2004). Phylogenetic analyses placed the RiCNE isolate within the Torix group, a sister lineage of the arthropod‐associated *Rickettsia*. We report the draft genome sequence for RiCNE, which represents the first sequenced genome of a *Rickettsia* belonging to the Torix group. Furthermore, we show Torix group *Rickettsia* are common in biting midges and, thus, represent a previously unrecognized component of the biology of this important vector group.

The draft genome of RiCNE provides valuable insights into the potential role of *Rickettsia* in midges and can further our understanding on the evolution of *Rickettsia* lifestyle and pathogenicity. The RiCNE draft genome shares many features with the previously sequenced *Rickettsia*, associated with genome reduction in the obligately intracellular genus. The genome size (∼ 1.5 Mb), the number of the protein‐coding genes (1352) and the coding density (80%) fell well within the range reported for *Rickettsia* (Merhej and Raoult, 2011; Gillespie *et al*., 2012).

Analysis of the metabolic potential of RiCNE shows a reduced biosynthetic and catabolic capacity typical of other *Rickettsia*, including absent or deficient pathways for glycolysis, nucleotide metabolism and amino‐acid biosynthesis. The blood feeding lifestyle of midges led us to particularly investigate the capacity for B vitamin synthesis, as recorded for *Wigglesworthia* symbionts in tsetse flies and *Wolbachia* in *Cimex* bedbugs (Snyder *et al*., 2010; Nikoh *et al*., 2014). However, with the exception of a reduced pathway for folate biosynthesis (also found in other *Rickettsia*), RiCNE lacks known pathways for the biosynthesis of cofactors and B‐vitamins.

A striking difference between the RiCNE genome and other arthropod‐associated *Rickettsia* is the presence of the complete set of genes encoding for the non‐oxidative branch of the PPP in RiCNE. The PPP is a major component of central metabolism in prokaryotes and eukaryotes (Stincone *et al*., 2015). The PPP is associated with both regulatory processes and biochemical functions, including carbon and redox homeostasis, response to oxidative stress and provision of precursors for nucleotide and amino acid biosynthesis. Notably, some parasites rely on the PPP to overcome the oxidative stress suffered during host invasion (Maugeri *et al*., 2003; Husain *et al*., 2012). Additionally, the non‐oxidative branch of the PPP in bacteria plays an essential role in the biosynthesis of lipopolysaccharides (LPS) by providing intermediates for the production of LPS precursors (Tzeng *et al*., 2002; Taylor *et al*., 2008). The biological role of the non‐oxidative PPP in RiCNE is unclear. Its presence in the *Rickettsia* endosymbiont of *I. multifilis* (Hydra group – ‘Megaira’) and its partial presence in *Occidentia massiliensis*, a sister species to *Orientia* isolated from a soft tick (Mediannikov *et al*., 2014), suggest that the non‐oxidative branch of the PPP has been independently lost in *Rickettsia* and *Orientia* lineages upon their transition to an arthropod host. Its absence from all other arthropod‐associated Rickettsiaceae may suggests specific functions to the lifestyle of Torix and Hydra group *Rickettsia* or to specific host microhabitats used by these symbionts (Fuchs *et al*., 2012). Alternatively, this can be suggestive of a relatively recent *Rickettsia* host shift to the midge host from a yet unknown ciliate host. Among the Rickettsiales, complete non‐oxidative PPP but absent oxidative PPP (as found in RiCNE) have been noted within the genera *Anaplasma*, *Ehrlichia* and *Neorickettsia* and the newly discovered member of the Midichloriaceae ‘*Candidatus* Jidaibacter acanthamoeba’, but in contrast, these pathways are incomplete in the genera *Wolbachia* and *Midichloria* (Fig. 4A). Our phylogenetic analysis suggests that the ancestor of the Rickettsiales had at least a partial PPP with a complete non‐oxidative phase, which was subsequently lost from certain lineages including most of the Rickettsiaceae. Further work should establish the degree to which the pathway is present in other Torix group *Rickettsia*, and the reasons for its loss more widely in the genus.

Rickettsiae have a complex surface structure, encoded by the presence of many of genes involved in LPS and peptidoglycan biosynthesis (Fuxelius *et al*., 2007). LPS are major components of the outer membrane in several Gram‐negative bacteria and constitute strong elicitors of the immune response both in insects and mammals (Raetz and Whitfield, 2002). Moreover, the capacity of intracellular, Gram‐negative, bacteria to modify their LPS components is essential for host immune evasion and host adaptation, influencing both pathogenicity and symbiosis (Li *et al*., 2012). Aside from the potential role of the PPP above in LPS biosynthesis, we found additional RiCNE‐specific genes associated with LPS and cell wall modification. Of note are the two paralogs of the lipid A 3‐*O*‐deacylase (*pagL*), a gene reported to be essential for establishing symbiosis in the nitrogen‐fixing endosymbiont *Rhizobium etli* (Brown *et al*., 2013). Recently the role of lipid A 3‐*O*‐deacylase in LPS remodelling and outer membrane vesicles (OMV) formation in bacteria has been reported (Elhenawy *et al*., 2016). Interestingly, OMVs have been reported to play essential roles in pathogenicity and symbiosis in several Gram‐negative bacteria. These roles include the delivery of virulence factors, modulation of host immune system, gut microbiota establishment and homoeostasis as well as horizontal DNA transfer (Ellis and Kuehn, 2010; Velimirov and Hagemann, 2011). Another example of a system associated with cell wall modification is the putative *N*‐acetylmuramoyl‐l‐alanine amidase (AmiD) gene encoding for a periplasmic lipoprotein involved in peptidoglycan recycling (Uehara and Park, 2007). It is noteworthy that aphids appear to have acquired horizontally an AmiD homologue, presumably from a rickettsial bacterium. This gene is highly upregulated specifically in the aphid bacteriocytes (the specialized host cells hosting its *Buchnera* symbiont), suggesting a potential role in bacteriocyte homeostasis and host–symbiont interaction (Nikoh *et al*., 2010).

Our second finding was that Torix group *Rickettsia* was found commonly across biting midges. Previous work on *Culicoides*, using conventional PCR to establish the presence of the heritable symbiont *Cardinium*, revealed interspecies infection rates ranging from 16% to 29% (Nakamura *et al*., 2009; Lewis *et al*., 2014; Mee *et al*., 2015). Thus, our PCR screen suggests that *Rickettsia* is the most common known symbiont of *Culicoides*, being present in 11 of 29 species tested (38%) and in 100% of specimens examined in 9 of the *Rickettsia* positive species. Hence, this *Rickettsia* clade represents an important associate found widely in *Culicoides* midges. It is noteworthy that our assessment of incidence is conservative, being based on a conventional PCR assay which will likely report false negatives for low titre infections.

The Torix group of *Rickettsia* has been recorded previously in an array of invertebrate species. Many of these species share ecological characteristics including an aquatic phase and predatory larval stages (e.g., midges, diving beetles, leeches and crane flies) (see Fig. 2). Others are notable for hematophagy (e.g., biting midges, leeches and sandflies). Moreover, no secondary associations with vertebrate hosts or pathogenicity have been associated so far with this *Rickettsia* group. Given the scarcity of available multilocus sequence data within Torix group, it is unclear whether the midge *Rickettsia* forms a monophyletic assemblage. More sequence data from other Torix *Rickettsia* will be needed to increase the phylogenetic resolution and determine the degree of relatedness among Torix Rickettsia strains. Nevertheless, our results support the hypothesis that Torix *Rickettsia* is a dominant taxon among invertebrates with aquatic life stages (Fig. 2).

The impact of the *Rickettsia* on host biology is uncertain. *Rickettsia* infections are known to be associated with a variety of reproductive manipulations of their host (reproductive parasitisms), including male‐killing in ladybird beetles (Werren *et al*., 1994) and parthenogenesis induction in parasitoids (Hagimori *et al*., 2006; Giorgini *et al*., 2010). However, equal likelihood of male and female midges being infected indicates sex ratio distortion is unlikely to be a phenotype for the *Rickettsia* in midges. Further to this, *Rickettsia* represents an obligate symbiont in book lice (*Liposcelis bostrychophila*) required for egg production (Perotti *et al*., 2006). However, the sporadic distribution of Rickettsia across subgenera suggests a lack of co‐speciation making it unlikely that the host requires symbiont presence for its function. Overall, the data suggests the Torix group *Rickettsia* identified in this study may have some facultative benefit to their host. Indeed, *Rickettsia* from this clade has been linked with a fitness benefit (increased body size) in leeches (Kikuchi and Fukatsu, 2005).

The strong tropism of *Rickettsia* bacteria for the midge oocytes unambiguously supports a vertical transmission route commonly seen in endosymbionts. This result also gives an indication of the likely routes driving *Rickettsia* to fixation within most of the populations in this study. There are a few routes that drive infection to fixation; combined horizontal and vertical transmission (Perlman *et al*., 2006), cytoplasmic incompatibility or non‐frequency‐dependent benefits combined with high fidelity maternal transmission and finally combined paternal and maternal transmission. The latter of these was described for the first time in Torix *Rickettisa* infecting leaf hoppers (*Nephotettix cincticeps*) (Watanabe *et al*., 2014). A peculiarity of note is the detection of coexisting infected and uninfected individuals in *C. impunctatus* populations, a scenario contrary to the more common fixed infections observed in this study. However, low titre infections cannot be ruled out (Mee *et al*., 2015). Alternative explanations for this difference are that the strain in *C. impunctatus* has a different role in its host in comparison to the other isolates at fixation in midges. In fact, the divergence of the *omp* gene in *C. impunctatus* (Supporting Information Fig. S6), one of the surface antigen coding genes, suggests possible differences in host specificity. *Rickettsia* surface antigens have previously been identified to be evolving under positive selection and may have key roles in host adherence and infiltration (Blanc *et al*., 2005). A major research effort for the future lies in identifying the impact of *Rickettsia* on host biology.

In conclusion, we have identified a common but neglected association between *Rickettsia* and biting midges and have described its unique genetic properties. Given the importance of biting midges as vectors, two key areas of future research are to establish the impact of *Rickettsia* presence on vector competence and on vector dispersal. Symbionts may reduce vector competence (as for *Wolbachia* in *Aedes aegypti*), increase it (as for *Rickettsia* in *Bemisia tabaci*) or have no impact. *Rickettsia* infections are also known to affect host dispersal tendencies, with Torix *Rickettsia*‐infected spiders showing lower motivation for dispersal (Goodacre *et al*., 2009). Symbiont impact on either of these characteristics would significantly alter the local and spatial spread of vector‐borne infections, thus pressingly deserve attention.

## Experimental procedures

### Genome sequencing, assembly and annotation

Genomic DNA from *C. newsteadi* N5 was extracted from single individuals using the QIAGEN DNAeasy™ Blood & Tissue Kit following the protocol for purification of total DNA from Insects. Equal concentrations of DNA from three individuals was pooled and used to construct a 500 bp paired‐end library (Illumina TruSeq Nano) that was sequenced on 1/3 lane of a HiSeq2500 platform at the Centre for Genomic Research (CGR), University of Liverpool, with 2 × 125 bp paired reads.

Quality assessment and filtering of the Illumina reads were performed using FastQC (Andrews, 2016) and FastX‐Toolkit (Gordon, 2010). A preliminary assembly was performed using SPAdes version 3.7.0 (Nurk *et al*., 2013) with k‐mer sizes 21, 33, 55 and 77 under ‘careful’ mode and a coverage cutoff of 5. Identification and filtering of putative symbiont contigs was performed by visualizing the data in taxon‐annotated GC‐coverage plots using Blobtools (Kumar *et al*., 2013; Laetsch, 2016) and TBLASTX searches against a local *Rickettsia*‐genomic database . *Rickettsia* contigs were extracted and any host contamination was removed by BLASTX searches against the non‐redundant protein database (NR). *Rickettsia*‐specific reads were retrieved using Bowtie2 (Langmead and Salzberg, 2012) and samtools (Li *et al*., 2009) and re‐assembled de novo with SPAdes assembler (k‐mer sizes: 21, 33, 55 and 77, ‘careful’ mode). The final assembly produced 224 contigs ≥ 500 bp, which were subjected to a final decontamination step removing only four contigs which had strong similarities to Enterobacteriaceae and lower than average coverage. Assembly errors were assessed using REAPR software (Hunt *et al*., 2013) (Supporting Information) and a final scaffolding was performed with SSPACE (Boetzer *et al*., 2011) with the following parameters, *k =* 5, *a =* 0.5 and *n =* 15.

The draft genome of the *Rickettsia* symbiont from *C. newsteadi* (RiCNE) was annotated using Prokka software v.1.12 (Seemann, 2014) (Supporting Information), and completeness was assessed using BUSCO v.2 based on 148 single‐copy universal bacterial markers (Simão *et al*., 2015). COG functional categories were assigned using the eggNOG 4.5 database (Huerta‐Cepas *et al*., 2016), and Pfam domains were predicted using InterProScan 5 (Jones *et al*., 2014). We evaluated the metabolic potential of RiCNE genome using the Metabolic and Physiological Potential Evaluator (MAPLE‐2.1.0) based on the calculation of the KEGG‐defined module completion ratio (MCR) (Takami *et al*., 2016). These results were compared with other *Rickettsia* from major *Rickettsia* groups (Belli, Adalia, Scapularis, Transitional, Typhus and Spotted Fever) as well as other members of the order Rickettsiales including the genera *Orientia*, *Wolbachia*, *Anaplasma*, *Ehrlichia* and *Midichloria*. Three known nutritional mutualists (*Wigglesworthia*, *Buchnera* and *Riesia*) were also included in the analyses.

### Ortholog identification and phylogenomic analyses

Identification of orthologous gene clusters (Orthogoups) was performed using OrthoFinder method (Emms and Kelly, 2015) on a dataset of 84 publicly available *Rickettsia* genomes as well as two *Orientia tsutsugamushi* strains (outgroup) (Supporting Information Table S2). In order to avoid inconsistencies arising from different annotation practices, all *Rickettsia* genomes used were re‐annotated using Prokka software as described above. A set of 189 single‐copy core orthogroups were selected (Supporting Information Table S10) and automatically aligned with MAFFT v7 (Katoh and Standley, 2013) using default settings. For phylogenetic analyses, a super‐matrix was generated by concatenating the protein alignments of the 189 single‐copy core genes and subsequently trimmed with trimAl version 1.4 (Capella‐Gutiérrez *et al*., 2009) using the ‘automated’ option. Phylogenetic relationships were reconstructed using maximum likelihood. The best protein model and substitution matrix was selected using ProtTest version 3.4.2 (Darriba *et al*., 2011) and maximum likelihood (ML) phylogeny were inferred with RAxML version 8.2.8 (Stamatakis, 2014) using 100 rapid bootstrap replicates under the PROTGAMMAILG model.

### Culicoides collection identification and DNA extractions

Overall, 414 specimens of 29 *Culicoides* species were collected using light traps from May 2007 to July 2016 across sites spanning France, South Africa, Sweden and the UK (Table 1 and Supporting Information Table S6). Sampled species included both vectors and non‐vectors of BTV. All midge specimens were stored in 70% ethanol for preservation before being sexed and separated morphologically down to the species level using relevant keys (Downes and Kettle, 1952; Campbell and Pelham‐Clinton, 1960; Delécolle, 1985; Meiswinkel, 1994). Morphological identification was confirmed by sequencing a fragment of the mitochondrial cytochrome c oxidase subunit 1 (COI) barcode (Pagès *et al*., 2009; Ander *et al*., 2013; Nielsen and Kristensen, 2015). DNA extractions were prepared based on the protocol of Ander *et al*. (2013) and details are presented in the Supporting Information. The *COI* gene fragment was amplified using different universal primer sets (Folmer *et al*., 1994; Dallas *et al*., 2003) and sequenced through the Sanger method by GATC Ltd. Samples that did not amplify were deemed to contain low quality DNA and were removed from further analysis.

### PCR screening for *Rickettsia* and analysis of strain relatedness

Presence of *Rickettsia* was initially assessed by PCR assay using *Rickettsia‐*specific primers designed to amplify a 320‐bp region of the *omp* (17 kDa surface antigen precursor) gene (Supporting Information Table S11). Cycling conditions were as follows: initial denaturation at 95°C for 5 min, followed by 35 cycles of denaturation (94°C, 30 s), annealing (54°C, 30 s), extension (72°C, 120 s), and a final extension at 72°C for 7 min. Amplicons identified by gel electrophoresis were subsequently purified enzymatically (ExoSAP) and sequenced (GATC Biotech AG, Konstanz, Germany).

Based on previous studies (Fournier *et al*., 2003; Weinert *et al*., 2009a; Li *et al*., 2010; Machtelinckx *et al*., 2012; Santibáñez *et al*., 2013) that profile *Rickettsia* diversity, the *16S rRNA*, g*ltA* (Citrate synthase), c*oxA* (cytochrome oxidase) and a*tpA* (ATP synthase) genes were chosen as indicators of genetic relatedness between isolates. With the consideration that these housekeeping loci may be too conserved to resolve recently diverged strains, the *omp* gene was included to allow higher resolution in typing, alongside an inference of selection pressure due to the divergent nature of antigen genes compared to housekeeping genes.

Primers to amplify these loci (Supporting Information Table S11) were designed on conserved regions based on *RiCNE* and available complete gene sequences so that they could amplify across several *Rickettsia* groups but would not cross amplify any alpha‐proteobacteria outgroups. All PCR amplifications were performed as described above. Sanger sequencing through both strands allowed for the clarification of ambiguous base calls as well as giving greater sequence coverage at individual loci. Raw sequences were edited in UGENE (Okonechnikov *et al*., 2012) and alignments for each locus were generated in MEGA6 using the ClustalW algorithm (Tamura *et al*., 2013).

A profile of each locus was constructed by calculating GC content, selective pressure (*K*_a_/*K*_s_), nucleotide diversity per site (*π*) and the percentage of variable sites using DNAsp v5 (Librado and Rozas, 2009). As phylogenetic inferences can be complicated by recombination, the presence of intragenic recombination was investigated using the program RDP v4 (Martin *et al*., 2015). To this end, the MaxChi algorithm was utilised with the following criteria to assess a true recombination positive: a *p* value of < 0.01, sequences were considered linear with 1200 permutations being performed. Recombination events detected by the programme were visually inspected for congruency between the recombinant and putative parent strains to confirm a true positive. As an additional aid, genetic divergence at each locus was determined using pairwise divergence. The *omp* locus was also assessed for evidence of diversifying selection at the gene level via pairwise non‐synonymous/synonymous rate ratio analysis (*K*_a_/*K*_s_ ratio).

Similar to multilocus sequence typing (MLST) convention, all unique genotypes were designated allele numbers (used as a unique identifier) which, when combined at all loci, produce an allelic profile (Maiden *et al*., 1998). Aside from identifying specific isolates, this multigenic approach also allows for clonal complexes to be identified (conventionally allelic profiles which are identical at three or more loci). Allelic profiles and complexes were designated based on Unweighted Pair Group Method with Arithmetic Mean (UPGMA) cluster analysis and visualised as a minimum spanning tree (MST) implemented by Bionumerics v7 (Applied Maths, Austin, TX, USA).

### Phylogenetic analyses

The phylogenetic position of the midge *Rickettsia* within the *Rickettsiaceae* was first assessed using the *16S rRNA* gene sequence. Briefly, sequences of *16S rRNA* from selected *Rickettsia* genomes used for the phylogenomic analyses (Bellii, Canadensis and Typhus groups) were extracted and combined with sequences from the Hydra and Torix Rickettsia groups (Weinert *et al*., 2009a) obtained from GenBank. All sequences were aligned using SSU‐ALIGN software (Nawrocki, 2009) and unambiguously aligned columns were automatically selected by ssu‐mask program. A Bayesian phylogeny was estimated with MrBayes v3.2.6 (Ronquist *et al*., 2012) under the GTR + G + I model. Two independent runs were carried out for 1,000,000 generations with sampling every 100 generations using four Markov chains. The first 25% of the samples were discarded as burn‐in. Alternative phylogenetic hypotheses were tested using constrain tree searches and the Shimodaira–Hasegawa (SH) test as implemented in RAxML version 8.2.8. The ML phylogeny for the *omp* gene was estimated with RaxML version 8.2.8 (Stamatakis, 2014) using 100 rapid bootstrap replicates under the GTR + G model of nucleotide substitutions. Single protein phylogenies of the PPP were estimated with Bayesian analyses using a mixed model of amino‐acid substitutions (two runs of 1,500,000 generations with sampling every 100 generations using four Markov chains). Additional ML analyses were inferred with RAxML version 8.2.8 (Stamatakis, 2014) using 100 rapid bootstrap replicates under the PROTGAMMAAUTO model optimization setting. Finally, trees were drawn using the iTOL (Letunic and Bork, 2007) and EvolView (He *et al*., 2016) online tree annotation and visualization tools.

### Fluorescent *in situ* hybridisation

Live nulliparous female *C. impunctatus* were collected from Kielder, UK and ovaries were dissected in 70% ethanol. Samples were fixed overnight in Carnoy's solution (chloroform:ethanol:glacial acetic acid, 6:3:1) and decolorized with 6% H_2_O_2_ in ethanol for 1 h. Hybridisation was performed overnight in hybridisation buffer (20 mM Tris‐HCl, pH 8.0, 0.9 M NaCl, 0.01% sodium dodecyl sulfate, 30% formamide) containing 10 pmol/ml of the *Rickettsia* specific probe [5′‐CCATCATCCCCTACTACA‐(ATTO 633)‐3′] adapted from Perotti *et al*. (2006). After hybridisation, the samples were thoroughly washed twice in hybridisation buffer (without the probe) and slide mounted in Vectashield with DAPI (Vector Laboratories) and viewed under a Zeiss LSM 880 BioAFM confocal microscope. The specificity of the detection and any autofluorescent properties of midge tissue was assessed using *Rickettsia*‐free midges (*Culicoides nubeculosus*; Pirbright Institute) as negative controls.

### Nucleotide sequence accession numbers

Raw reads and the RiCNE draft genome assembly have been submitted to the DDBJ/EMBL/GenBank database under the BioProject accession number PRJNA376033 (WGS project: MWZE00000000). COI barcodes and sequences generated for individual *Rickettsia* loci in this study were deposited in GenBank under deposition numbers KY765346‐KY765408, KY777722‐KY777733 and KY778697‐KY778698.

## Author contributions

Acquisition, analysis and interpretation of the data were undertaken by JP and SS, as well as drafting of the manuscript. GH and MB assisted in the conception and design of the study, in addition to critical revision of the manuscript. CG and MA aided in the collection and identification of midge specimens and the critical revision of the manuscript.

## Supporting information

Additional Supporting Information may be found in the online version of this article at the publisher's web‐site:

**Fig. S1.** Taxon annotated GC‐coverage plots. (A) Primary genome assembly of *Culicoides newsteadi* N5. (B) Postfiltering against a local database containing all available (complete and draft) *Rickettsia* genomes.

**Fig. S2.** BUSCO completeness assessment results for RiCNE draft genome in relation to selected complete *Rickettsia* genomes. The results are based on the presence or absence of 148 single‐copy universal bacterial markers. BUSCO notation: complete (C), single‐copy (S), duplicated (D), fragmented (F) and missing (M).

**Fig. S3.** Circular representation of the RiCNE draft genome. For visualization purposes, the 193 scaffolds were concatenated into a pseudomolecule. Alternating gray and white strips indicate scaffold borders. Inwards, the first, second and third circles are color‐coded according to the eggNOG functional categories and represent: (a) the complete RiCNE protein‐coding genes (CDSs), (b) CDSs universally present in all Rickettsiacea genomes used in this study and (c) RiCNE unique CDSs respectively. The fourth circle shows the clusters of the T4SS (red) and conjugation (purple) genes. The first line plot represents the genome coverage. An orange line indicates the average coverage of the draft assembly (∼ 76×). The asterisks indicate the two scaffolds containing the conjugation genes represented by 2–3× higher than average coverage indicating possible multiple copies. The innermost line plot represents the GC% coverage calculated based on a 1 kb sliding window. The circular plot was generated with Circos v0.69 (Krzywinski *et al*., 2009).

**Fig. S4.** Cladogram of the complete core‐genome phylogeny. Phylogenomic placement of the *Rickettsia* endosymbiont of *C. newsteadii* were inferred using maximum likelihood (RaxML, model: Lag + G + I) from the concatenated protein alignments of 189 single copy ortholog genes. Support values are based on 100 rapid bootsrap replicates.

**Fig. S5.** Individual trees for the pentose phosphate pathway (PPP) proteins. Tree topology and posterior probabilities were inferred with MrBayes using a mixed model of amino acid substitution. The trees were midpoint rooted.

**Fig. S6.** Maximum likelihood phylogeny of the *omp* gene. The tree topology was estimated using RaxML and the GTR + G model of nucleotide substitutions. Support values are based on 1000 rapid bootstrap replicates. The tree was midpoint rooted.

**Table S1.** Functional annotation of RiCNE draft genome including Interproscan results against Pfam and eggNOG results.

**Table S2.** Publicly available Rickettsiaceae genomes used in this study for ortholog identification and phylogenomic analysis.

**Table S3.** Genes encoding for the P‐T4SS and the tra conjugative DNA‐transfer element in RiCNE genome.

**TableS4.** RiCNE unique genes.

**Table S5.** RiCNE unique genes putatively associated with host invasion and host–microbe interactions.

**Table S6.** omp conventional PCR assay results for *Rickettsia*‐negative *Culicoides* species under study, given by subgenus, species, location, date and sex. ^a^ *Culicoides newsteadi* haplotype N1 designated by Ander *et al*. (2013). ^b^ *Culicoides newsteadi* N6 previously undesignated.

**Table S7.** Genetic characteristics of housekeeping and *omp* alleles.

**Table S8.** *Rickettsia* strains recovered from *Culicoides* midges, with allelic profiles; strains sharing the same allelic profiles at all five loci were designated as a single strain. NA: non amplifiable.

**Table S9.** Pairwise divergence at individual loci between strains from clonal complex 2 showing remarkable divergence in the ATPase allele compared to the average of all strains, most likely as a result of a recombination event.

**Table S10.** Core genes used for phylogenomic analysis.

**Table S11.** Housekeeping and *omp* gene primer attributes.
