# Supplementary material for: Torix group Rickettsia are widespread in Culicoides biting midges (Diptera: Ceratopogonidae), reach high frequency and carry unique genomic features
Source: Environ Microbiol. 2017 Sep 18;19(10):4238–55. doi: 10.1111/1462-2920.13887 (PMC5656822; doi:10.1111/1462-2920.13887)
Supplement: Supplementary file 1 — Fig. S1. Taxon annotated GC‐coverage plots. (A) Primary genome assembly of Culicoides newsteadi N5. (B) Postfiltering against a local database containing all available (complete and draft) Rickettsia genomes. [file EMI-19-4238-s001.pdf]

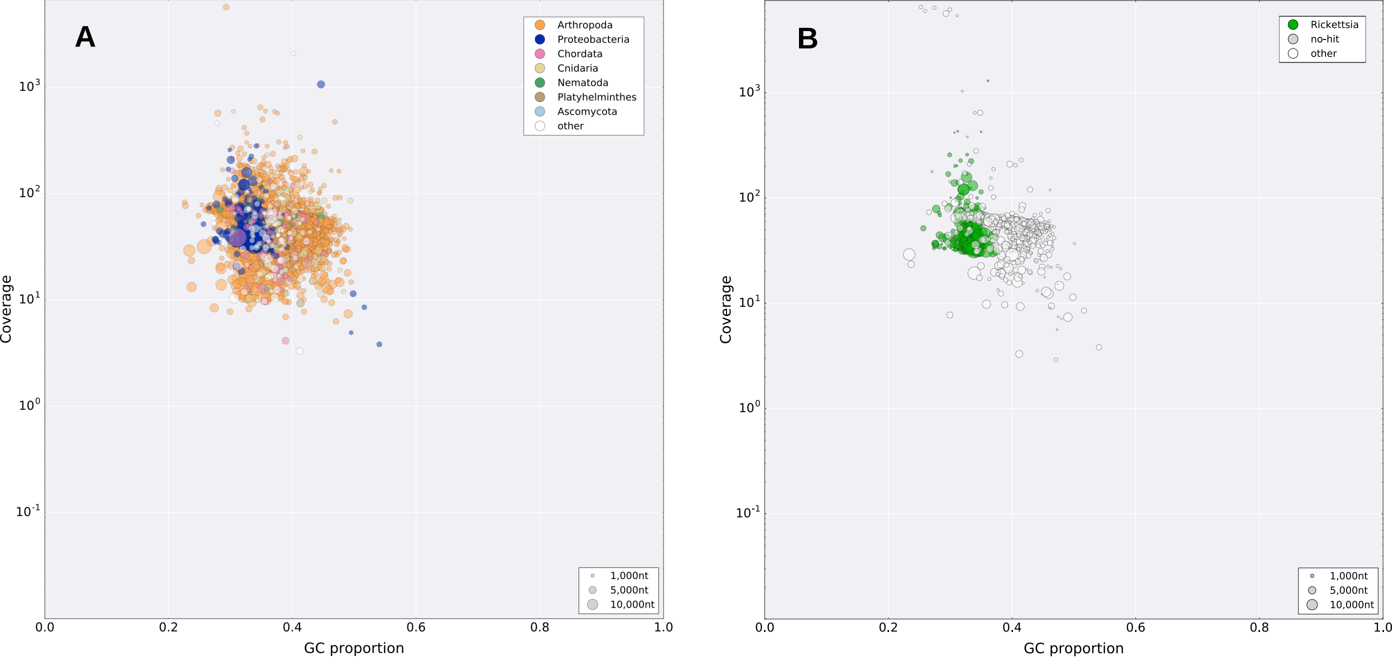

Figure S1. Taxon annotated GC-coverageplots. A) Primary genome assembly of Culicoides newsteadi N5. B) post filtering against a local database containing all available (complete and draft) Rickettsia genomes.
