# Supplementary material for: Torix group Rickettsia are widespread in Culicoides biting midges (Diptera: Ceratopogonidae), reach high frequency and carry unique genomic features
Source: Environ Microbiol. 2017 Sep 18;19(10):4238–55. doi: 10.1111/1462-2920.13887 (PMC5656822; doi:10.1111/1462-2920.13887)
Supplement: Supplementary file 2 — Fig. S2. BUSCO completeness assessment results for RiCNE draft genome in relation to selected complete Rickettsia genomes. The results are based on the presence or absence of 148 single‐copy universal bacterial markers. BUSCO notation: complete (C), single‐copy (S), duplicated (D), fragmented (F) and missing (M). [file EMI-19-4238-s002.pdf]

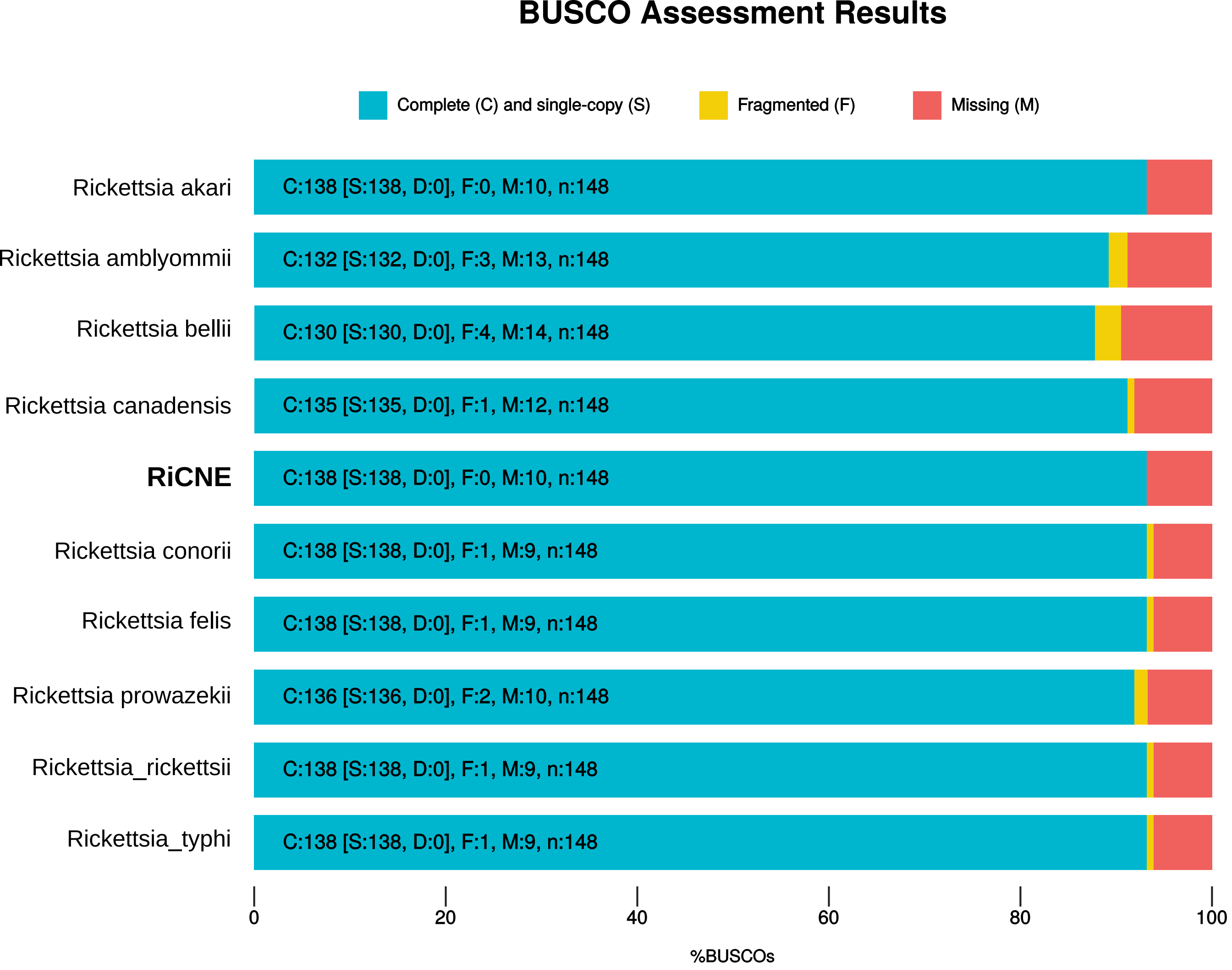

Figure S2. BUSCO completeness assessment results for RiCNE draft genome in relation to selected complete Rickettsia genomes. The Results are based on the presence or absence of 148 single-copy universal bacterial markers. BUSCO notation: complete (C), single-copy (S), duplicated (D), fragmented (F) and missing (M).
