# Supplementary material for: Torix group Rickettsia are widespread in Culicoides biting midges (Diptera: Ceratopogonidae), reach high frequency and carry unique genomic features
Source: Environ Microbiol. 2017 Sep 18;19(10):4238–55. doi: 10.1111/1462-2920.13887 (PMC5656822; doi:10.1111/1462-2920.13887)
Supplement: Supplementary file 3 — Fig. S3. Circular representation of the RiCNE draft genome. For visualization purposes, the 193 scaffolds were concatenated into a pseudomolecule. Alternating gray and white strips indicate scaffold borders. Inwards, the first, second and third circles are color‐coded according to the eggNOG functional categories and represent: (a) the complete RiCNE protein‐coding genes (CDSs), (b) CDSs universally present in all Rickettsiacea genomes used in this study and (c) RiCNE unique CDSs respectively. The fourth circle shows the clusters of the T4SS (red) and conjugation (purple) genes. The first line plot represents the genome coverage. An orange line indicates the average coverage of the draft assembly (∼ 76×). The asterisks indicate the two scaffolds containing the conjugation genes represented by 2–3× higher than average coverage indicating possible multiple copies. The innermost line plot represents the GC% coverage calculated based on a 1 kb sliding window. The circular plot was generated with Circos v0.69 (Krzywinski et al., 2009). [file EMI-19-4238-s003.pdf]

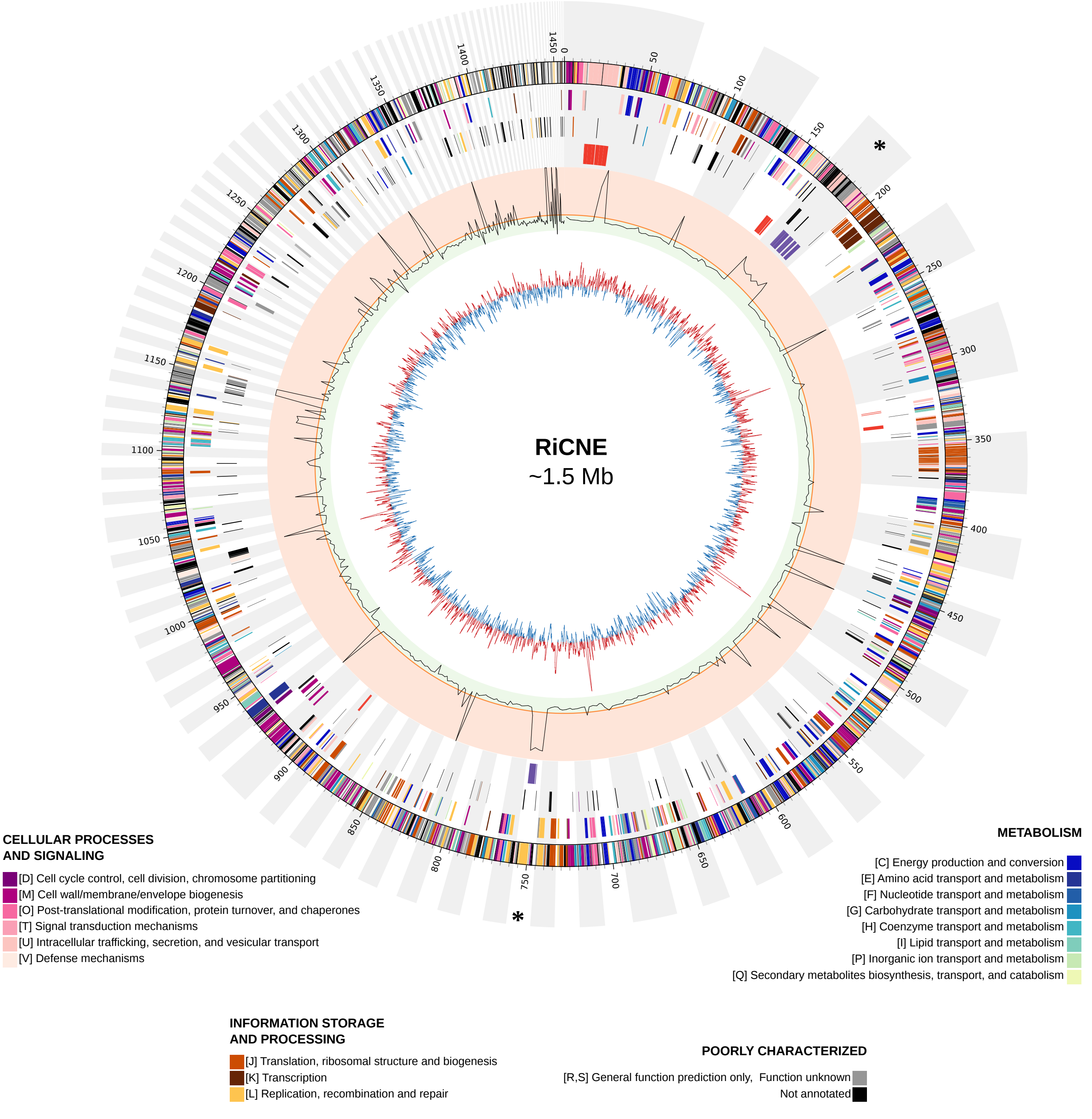

Figure S3. Circular representation of the RiCNE draft genome. For visualization purposes the 193 scaffolds were concatenated into a pseudomolecule. Alternating gray and white strips indicate scaffold borders. Inwards, the first, second and third circles are color-coded according to the eggNOG functional categories and represent: a) the complete RiCNE protein-coding genes (CDSs), b) CDSs universally present in all Rickettsiacea genomes used in this study and c) RiCNE unique CDSs respectively. The fourth circle shows the clusters of the T4SS (red) and conjugation (purple) genes. The first line plot represents the genome coverage. An orange line indicate the average coverage of the draft assembly (~76X). The asterisks indicate the two scaffolds containing the conjugation genes represented by 2-3x higher than average coverage indicating possible multiple copies. The innermost line plot represents the GC% coverage calculated based on a 1kb sliding window. The circular plot was generated with Circos v0.69 (Krzywinski et al., 2009).
