# Supplementary material for: Torix group Rickettsia are widespread in Culicoides biting midges (Diptera: Ceratopogonidae), reach high frequency and carry unique genomic features
Source: Environ Microbiol. 2017 Sep 18;19(10):4238–55. doi: 10.1111/1462-2920.13887 (PMC5656822; doi:10.1111/1462-2920.13887)
Supplement: Supplementary file 5 — Fig. S5. Individual trees for the pentose phosphate pathway (PPP) proteins. Tree topology and posterior probabilities were inferred with MrBayes using a mixed model of amino acid substitution. The trees were midpoint rooted. [file EMI-19-4238-s005.pdf]

rpe

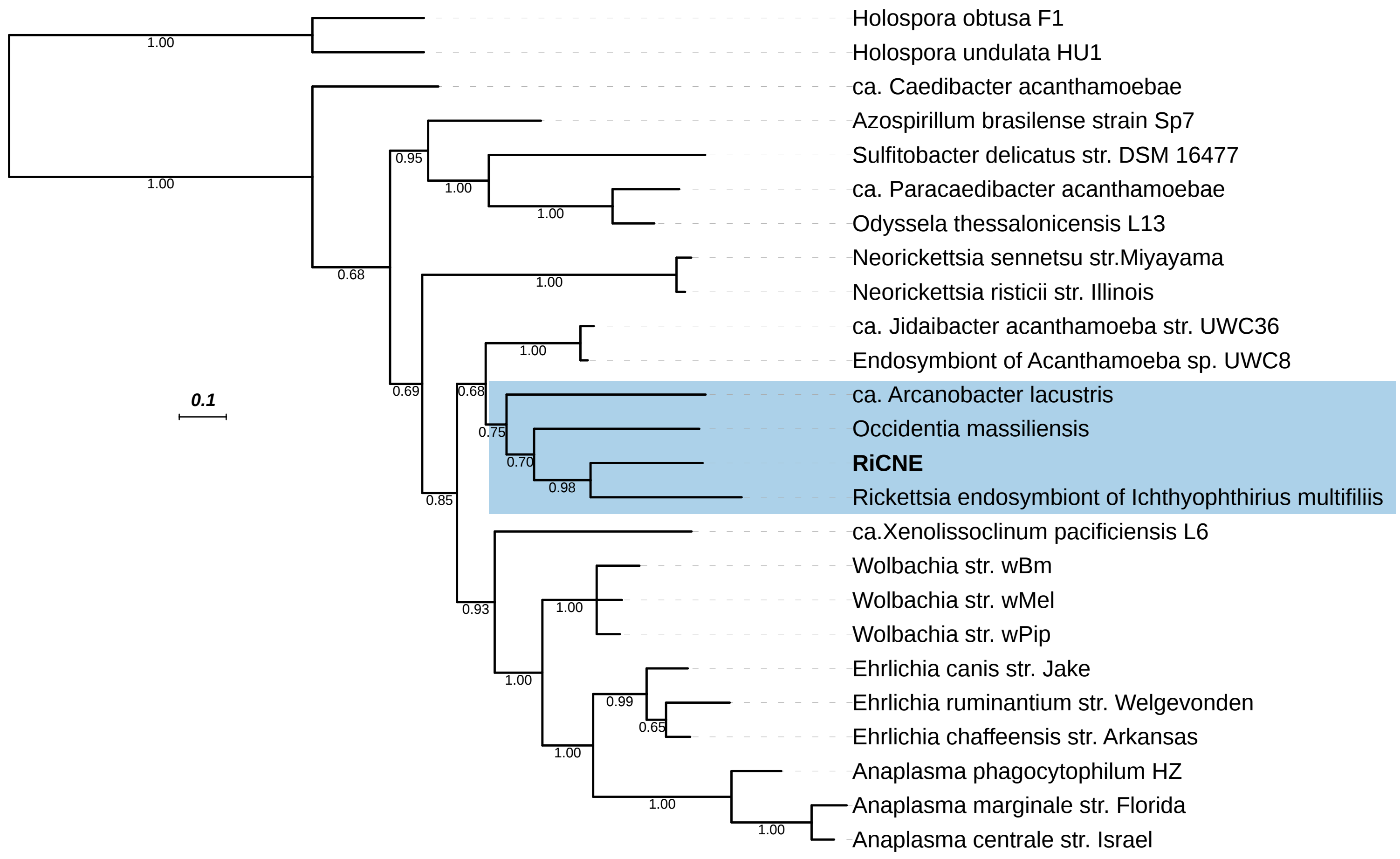

Holospora obtusa F1
Holospora undulata HU1
ca. Caedibacter acanthamoebae
Azospirillum brasilense strain Sp7
Sulfitobacter delicatus str. DSM 16477
ca. Paracaedibacter acanthamoebae
Odyssela thessalonicensis L13
Neorickettsia sennetsu str.Miyayama
Neorickettsia risticii str. Illinois
ca. Jidaibacter acanthamoeba str. UWC36
Endosymbiont of Acanthamoeba sp. UWC8
ca. Arcanobacter lacustris
Occidentia massiliensis
RiCNE
Rickettsia endosymbiont of Ichthyophthirius multifiliis
ca.Xenolissoclinum pacificiensis L6
Wolbachia str. wBm
Wolbachia str. wMel
Wolbachia str. wPip
Ehrlichia canis str. Jake
Ehrlichia ruminantium str. Welgevonden
Ehrlichia chaffeensis str. Arkansas
Anaplasma phagocytophilum HZ
Anaplasma marginale str. Florida
Anaplasma centrale str. Israel
0.1
1.00
1.00
0.95
1.00
1.00
0.68
1.00
1.00
0.69
0.68
0.75
0.70
0.98
0.85
0.93
1.00
1.00
0.99
0.65
1.00
1.00
1.00

tla

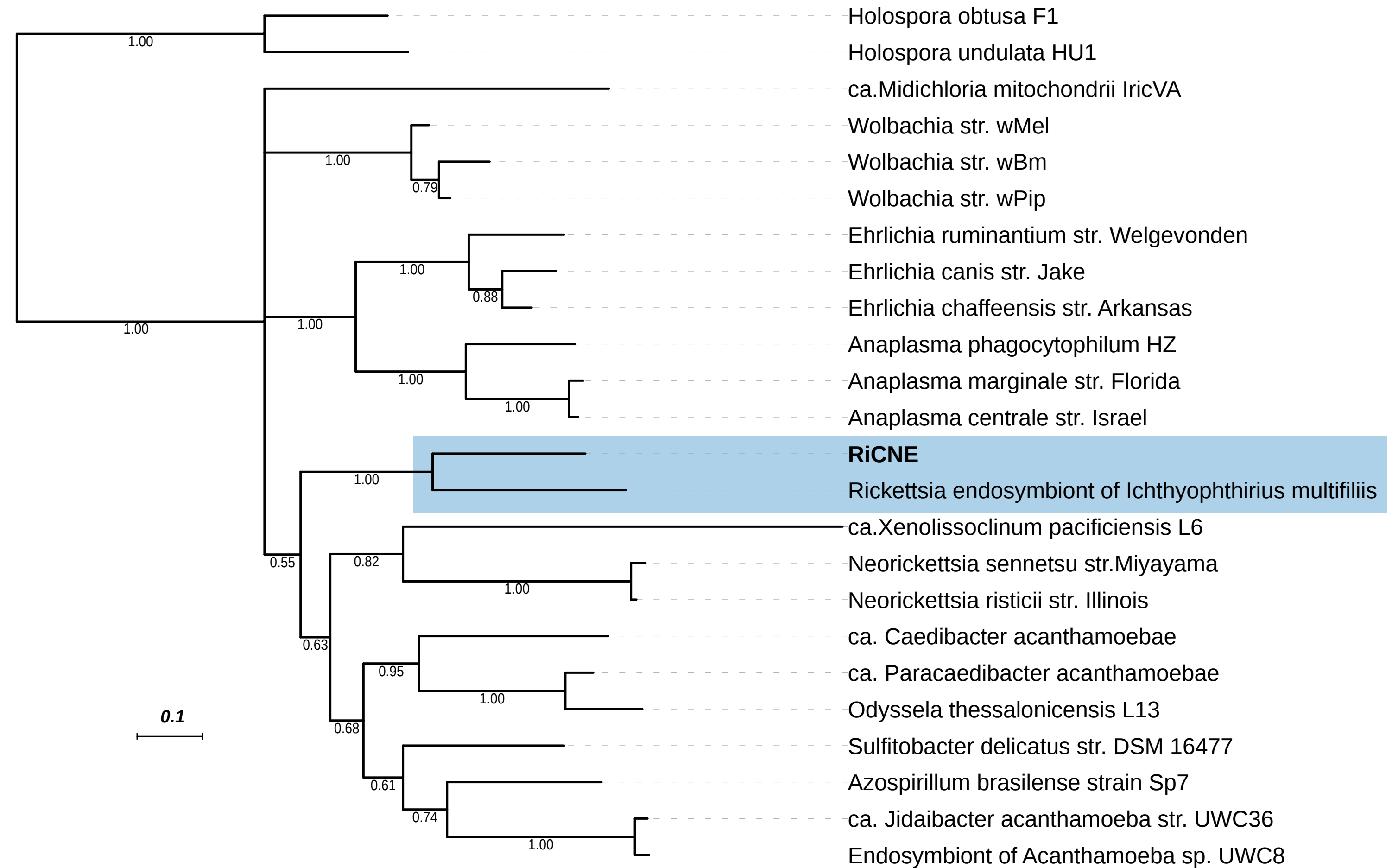

Holospora obtusa F1
Holospora undulata HU1
ca.Midichloria mitochondrii IricVA
Wolbachia str. wMel
Wolbachia str. wBm
Wolbachia str. wPip
Ehrlichia ruminantium str. Welgevonden
Ehrlichia canis str. Jake
Ehrlichia chaffeensis str. Arkansas
Anaplasma phagocytophilum HZ
Anaplasma marginale str. Florida
Anaplasma centrale str. Israel
RiCNE
Rickettsia endosymbiont of Ichthyophthirius multifiliis
ca.Xenolissoclinum pacificiensis L6
Neorickettsia sennetsu str.Miyayama
Neorickettsia risticii str. Illinois
ca. Caedibacter acanthamoebae
ca. Paracaedibacter acanthamoebae
Odyssela thessalonicensis L13
Sulfitobacter delicatus str. DSM 16477
Azospirillum brasilense strain Sp7
ca. Jidaibacter acanthamoeba str. UWC36
Endosymbiont of Acanthamoeba sp. UWC8
0.1
1.00
1.00
1.00
0.79
1.00
1.00
0.88
1.00
1.00
1.00
0.55
0.82
1.00
0.63
0.95
1.00
0.68
0.61
0.74
1.00

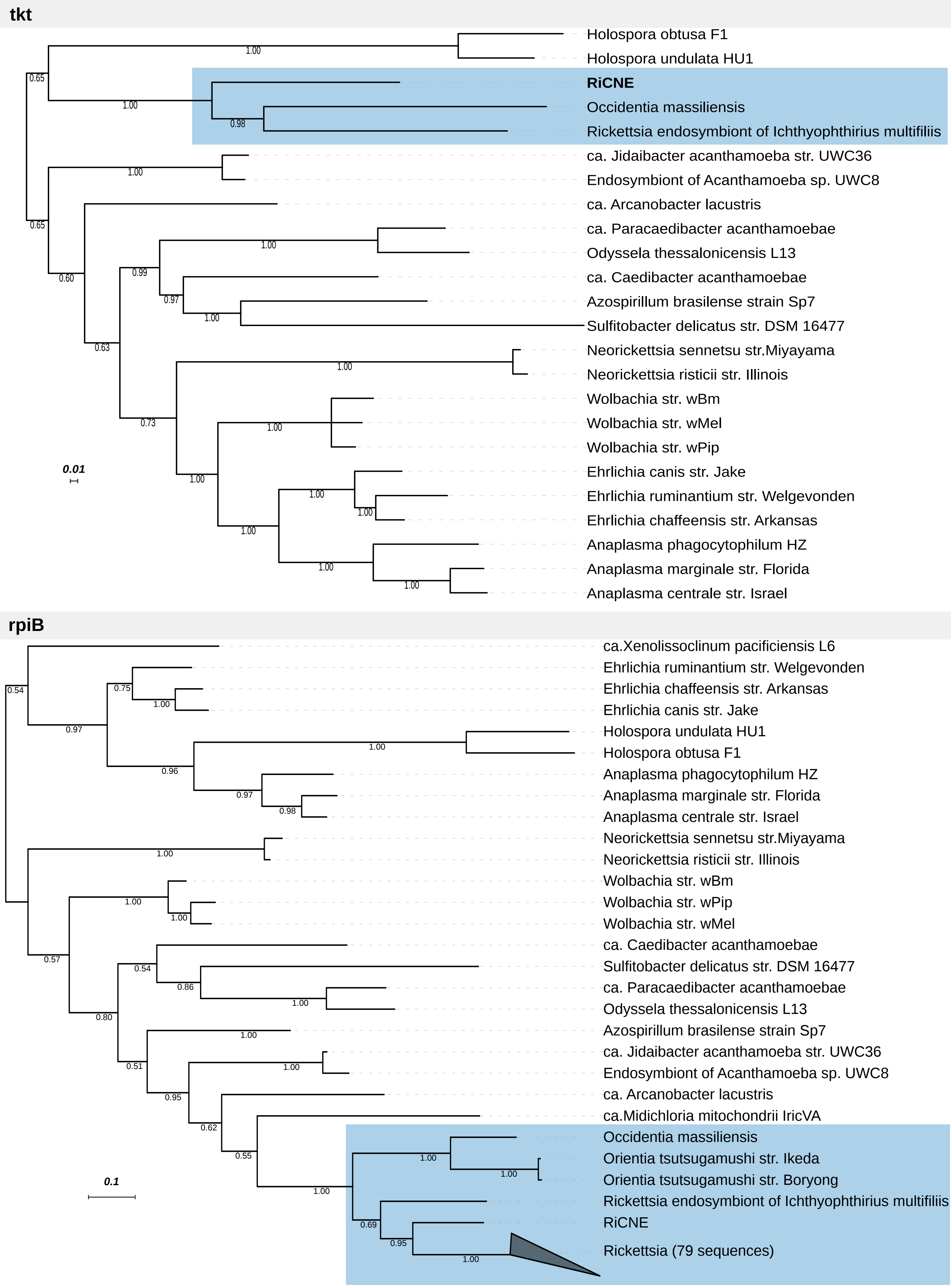

Figure S5. Individual trees for the Pentose Phosphate Pathway (PPP) proteins. Tree topology and posterior probabilities were inferred with MrBayes using a mixed model of amino acid substitution. The trees were midpoint rooted.
