# Supplementary material for: Torix group Rickettsia are widespread in Culicoides biting midges (Diptera: Ceratopogonidae), reach high frequency and carry unique genomic features
Source: Environ Microbiol. 2017 Sep 18;19(10):4238–55. doi: 10.1111/1462-2920.13887 (PMC5656822; doi:10.1111/1462-2920.13887)
Supplement: Supplementary file 6 — Fig. S6. Maximum likelihood phylogeny of the omp gene. The tree topology was estimated using RaxML and the GTR + G model of nucleotide substitutions. Support values are based on 1000 rapid bootstrap replicates. The tree was midpoint rooted. [file EMI-19-4238-s006.pdf]

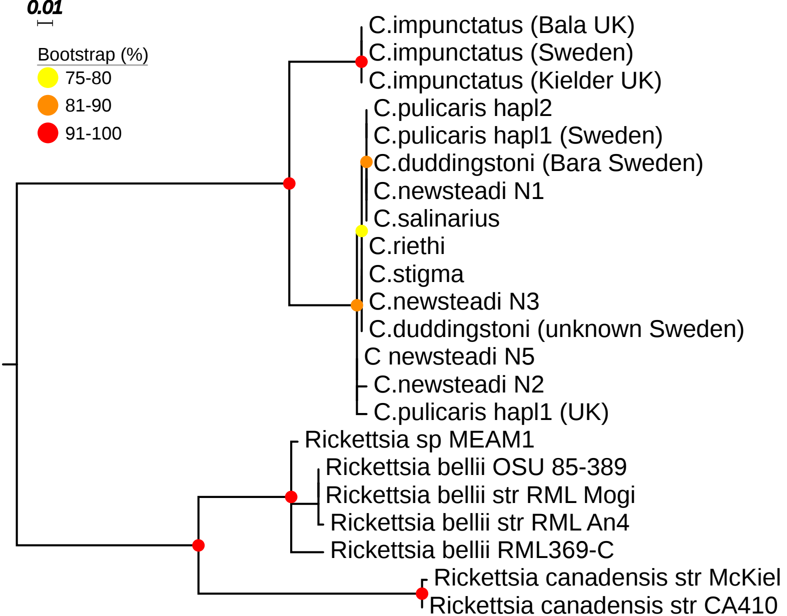

Figure S6. Maximum likelihood phylogeny of the omp gene. The tree topology was estimated using RaxML and the GTR+G model of nucleotide substitutions. Support values are based on 1000 rapid bootstrap replicates. The tree was midpoint rooted.
