# Supplementary material for: Torix group Rickettsia are widespread in Culicoides biting midges (Diptera: Ceratopogonidae), reach high frequency and carry unique genomic features
Source: Environ Microbiol. 2017 Sep 18;19(10):4238–55. doi: 10.1111/1462-2920.13887 (PMC5656822; doi:10.1111/1462-2920.13887)
Supplement: Supplementary file 12 — Table S6. omp conventional PCR assay results for Rickettsia‐negative Culicoides species under study, given by subgenus, species, location, date and sex. a Culicoides newsteadi haplotype N1 designated by Ander et al. (2013). b Culicoides newsteadi N6 previously undesignated. [file EMI-19-4238-s012.doc]

**Table S6.** *omp* conventional PCR assay results for *Rickettsia*-negative *Culicoides* species under study, given by subgenus, species, location, date and sex. *Culicoides newsteadi* haplotype N1a designated by Pagès *et al*. (2009), *Culicoides newsteadi* N6b previously undesignated. ^*^*C. newsteadi* haplotypes are defined as separate species as their *COI* barcodes have <81% identity between each other.

|  |  |  |  | **Proportion of *Rickettsia* positive samples**  **(n) [95% Confidence interval]** | |
| --- | --- | --- | --- | --- | --- |
| **Subgenus** | ***Culicoides* species** | **Location** | **Year of Collection** | **Females** | **Males** |
| Avaritia | *C. bolitinos* | Onderstepoort, South Africa | 2016 |  | 0 (2) [0-0.8] |
|  |  | Port Elizabeth, South Africa | 2014-2016 | 0 (18) [0-0.22] |  |
|  | *C. imicola* | Onderstepoort, South Africa | 2016 | 0 (23) [0-0.18] | 0 (9) [0-0.37] |
|  |  | Site 1, Corsica | 2015 | 0 (24) [0-0.17] |  |
|  |  | Site 2, Corsica | 2015 | 0 (16) [0-0.24] |  |
|  | *C. obsoletus* | Bara, Sweden | 2008 |  | 0 (8) [0-0.4] |
|  |  | Wirral, UK | 2012-2015 | 0 (14) [0-0.27] |  |
|  |  |  |  |  |  |
| Beltranmyia | *C. circumscriptus* | Unknown site, Sweden | 2009 | 0 (1) [0-0.95] |  |
|  | *C. sphagnumensis* | Axvalla, Sweden | 2009 | 0 (1) [0-0.95] |  |
|  |  |  |  |  |  |
| Culicoides | *C. brucei* | Onderstepoort, South Africa | 2016 | 0 (1) [0-0.95] |  |
|  | *C. grisescens* | Torsås, Sweden | 2008 | 0 (4) [0-0.6] |  |
|  | *C. newsteadi N1*^a*^ | Site 1, Corsica | 2015 | 0 (4) [0-0.6] |  |
|  | *C. newsteadi N6*^b*^ | Wolverhampton, UK | 2014 |  | 0 (1) [0-0.95] |
|  |  | Site 1, Corsica | 2015 | 0 (12) [0-0.30] |  |
|  |  | Site 2, Corsica | 2015 | 0 (3) [0-69] |  |
|  | *C. punctatus* | Wolverhampton, UK | 2014 | 0 (4) [0-0.6] |  |
|  |  | Wirral, UK | 2015 | 0 (23) [0-0.18] |  |
|  |  | Torsås, Sweden | 2008 | 0 (14) [0-0.27] |  |
|  |  | Unknown site, Sweden | 2008 |  | 0 (9) [0-0.37] |
|  |  |  |  |  |  |
| Oecacta | *C. clastrieri* | Unknown site, Sweden | 2009 |  | 0 (1) [0-0.95] |
|  | *C. festivipennis* | Uppsalla, Sweden | 2009 | 0 (1) [0-0.95] |  |
|  | *C. truncorum* | Torsås, Sweden | 2008-2009 | 0 (3) [0-0.69] | 0 (2) [0-0.8] |
|  |  |  |  |  |  |
| Remmia | *C. subshultzei* | Onderstepoort, South Africa | 2016 |  | 0 (5) [0-0.54] |
|  |  |  |  |  |  |
| Silvaticulicoides | *C. achrayi* | Axvalla, Sweden | 2008 | 0 (2) [0-0.8] |  |
|  | *C. subfascipennis* | Romakloster, Sweden | 2008 | 0 (8) [0-0.4] |  |
|  |  |  |  |  |  |
| Synhelea | *C. bedfordi* | Onderstepoort, South Africa | 2016 | 0 (1) [0-0.95] |  |
|  |  |  |  |  |  |
| Wirthomyia | *C. reconditus* | Unknown site, Sweden | 2007-2010 | 0 (3) [0-0.69] |  |
|  | *C. segnis* | Romakloster, Sweden | 2008 | 0 (11) [0-0.32] |  |
