# Supplementary material for: Torix group Rickettsia are widespread in Culicoides biting midges (Diptera: Ceratopogonidae), reach high frequency and carry unique genomic features
Source: Environ Microbiol. 2017 Sep 18;19(10):4238–55. doi: 10.1111/1462-2920.13887 (PMC5656822; doi:10.1111/1462-2920.13887)
Supplement: Supplementary file 13 — Table S7. Genetic characteristics of housekeeping and omp alleles. [file EMI-19-4238-s013.doc]

**Table S7.** Genetic characteristics of housekeeping and *omp* alleles.

| **Locus** | **Alleles** | **Nucleotide diversity/site (π)** | **Variable sites (%)** | **G+C content** | **K_a_/K_s_ average** | **Recombination (MaxChi *p*<0.01)** |
| --- | --- | --- | --- | --- | --- | --- |
| AtpA | 10 | 0.046 | 9.9 | 0.396 | 0.059 | Yes |
| CoxA | 6 | 0.012 | 4.0 | 0.372 | 0.074 | No |
| GltA | 7 | 0.013 | 3.5 | 0.364 | 0.035 | No |
| 16S | 8 | 0.002 | 0.9 | 0.511 | N/A | No |
| Omp | 6 | 0.018 | 10.7 | 0.423 | 0.317 | No |
