# Supplementary material for: Torix group Rickettsia are widespread in Culicoides biting midges (Diptera: Ceratopogonidae), reach high frequency and carry unique genomic features
Source: Environ Microbiol. 2017 Sep 18;19(10):4238–55. doi: 10.1111/1462-2920.13887 (PMC5656822; doi:10.1111/1462-2920.13887)
Supplement: Supplementary file 14 — Table S8. Rickettsia strains recovered from Culicoides midges, with allelic profiles; strains sharing the same allelic profiles at all five loci were designated as a single strain. NA: non amplifiable. [file EMI-19-4238-s014.doc]

**Table S8.** *Rickettsia* strains recovered from *Culicoides* midges, with allelic profiles; strains sharing the same allelic profiles at all five loci were designated as a single strain. NA=non amplifiable.

| **Species** | **Strain identity** | **Clonal complex** | **ATPase** | **COX** | **GLT** | **16S** | **omp** |
| --- | --- | --- | --- | --- | --- | --- | --- |
| *C. stigma* | A | 1 | 1 | 1 | 1 | 1 | 1 |
| *C. newsteadi* N3 | A | 1 | 1 | 1 | 1 | 1 | 1 |
| *C. riethi* | B | 1 | 1 | 1 | 1 | 3 | 1 |
| *C. newsteadi* N1 | C | 2 | 2 | 2 | 2 | 2 | 2 |
| *C. duddingstoni* (Bara, Sweden) | C | 2 | 2 | 2 | 2 | 2 | 2 |
| *C. pulicaris* haplotype 1 (Sweden) | D | 2 | 5 | 2 | 2 | 2 | 2 |
| *C. pulicaris* haplotype 2 (Corsica) | E | 2 | 7 | 2 | 2 | 2 | 2 |
| *C. salinarius* | F | 3 | 3 | 3 | 3 | 4 | 2 |
| *C. duddingstoni* (Unknown site, Sweden) | G | 4 | 4 | 2 | 4 | 5 | 1 |
| *C. newsteadi* N2 | H | 5 | 6 | 4 | 5 | 6 | 3 |
| *C. pulicaris* haplotype 1 (UK) | I | 6 | 8 | NA | 6 | 7 | 4 |
| *C. newsteadi* N5 | J | 7 | 9 | 5 | 7 | 8 | 5 |
| *C. impunctatus* | K | 8 | 10 | 6 | 8 | 9 | 6 |
