# Supplementary material for: Torix group Rickettsia are widespread in Culicoides biting midges (Diptera: Ceratopogonidae), reach high frequency and carry unique genomic features
Source: Environ Microbiol. 2017 Sep 18;19(10):4238–55. doi: 10.1111/1462-2920.13887 (PMC5656822; doi:10.1111/1462-2920.13887)
Supplement: Supplementary file 15 — Table S9. Pairwise divergence at individual loci between strains from clonal complex 2 showing remarkable divergence in the ATPase allele compared to the average of all strains, most likely as a result of a recombination event. [file EMI-19-4238-s015.doc]

**Table S9.** Pairwise divergence at individual loci between strains from clonal complex 2 showing remarkable divergence in the ATPase allele compared to the average of all strains, most likely as a result of a recombination event.

| **Species (Strain)** | **ATPase** | **COX** | **GLT** | **16S** | **OMP** |
| --- | --- | --- | --- | --- | --- |
| *C. pulicaris* haplotype 2 - *C. pulicaris* haplotype 1 (Sweden) (E-D) | 0.088 | 0 | 0 | 0 | 0 |
| *C. newsteadi* N1 - *C. pulicaris* haplotype 1 (Sweden) (C-D) | 0.088 | 0 | 0 | 0 | 0 |
| *C. newsteadi* N1 - *C. pulicaris* haplotype 2 (C-E) | 0.001 | 0 | 0 | 0 | 0 |
| **Average (all strains)** | **0.046** | **0.012** | **0.013** | **0.002** | **0.018** |
