# Supplementary material for: Torix group Rickettsia are widespread in Culicoides biting midges (Diptera: Ceratopogonidae), reach high frequency and carry unique genomic features
Source: Environ Microbiol. 2017 Sep 18;19(10):4238–55. doi: 10.1111/1462-2920.13887 (PMC5656822; doi:10.1111/1462-2920.13887)
Supplement: Supplementary file 17 — Table S11. Housekeeping and omp gene primer attributes. [file EMI-19-4238-s017.doc]

**Table S11.** Housekeeping and *omp* gene primer attributes.

| **Gene** | **Product** | **Primer name** | **Sequence (5’-3’)** | **Gene length (bp)** | **MLST fragment size (bp)** |
| --- | --- | --- | --- | --- | --- |
| AtpA^*^ | ATP synthase subunit alpha | RiAtpA327_F | GTCGGTAAAGCATTGCTTGGT | 1560 | 977 |
|  |  | RiAtpA1309_R | ATTGATCCTGCTTCAATA |  |  |
| CoxA^*^ | Cytochrome c oxidase, subunit I | RiCoxA317_F | ATAGGTGCACCGGATATGGC | 1569 | 1021 |
|  |  | RiCoxA1409_R | CCGATAGATGATACCATATTCCA |  |  |
| GltA^*^ | Citrate synthase | RiGltA405_F | GATCATCCTATGGCA | 1287 | 786 |
|  |  | RiGltA1193_R | TCTTTCCATTGCCCC |  |  |
| Omp^*^ | Outer membrane protein | Ri17kD_F | TCTGGCATGAATAAACAAGG | 471 | 319 |
|  |  | Ri17kD_R | ACTCACGACAATATTGCCC |  |  |
| 16S^$^ | 16S ribosomal RNA | Ri170_F | GGGCTTGCTCTAAATTAGTTAGT | 1511 | 1170 |
|  |  | Ri1500_R | ACGTTAGCTCACCACCTTCAGG |  |  |

^*^ This study.

^$^ Kuchler *et al*., 2009
