# Supplementary material for: Torix group Rickettsia are widespread in Culicoides biting midges (Diptera: Ceratopogonidae), reach high frequency and carry unique genomic features
Source: Environ Microbiol. 2017 Sep 18;19(10):4238–55. doi: 10.1111/1462-2920.13887 (PMC5656822; doi:10.1111/1462-2920.13887)
Supplement: Supplementary file 4 — Fig. S4. Cladogram of the complete core‐genome phylogeny. Phylogenomic placement of the Rickettsia endosymbiont of C. newsteadii were inferred using maximum likelihood (RaxML, model: Lag + G + I) from the concatenated protein alignments of 189 single copy ortholog genes. Support values are based on 100 rapid bootsrap replicates. [file EMI-19-4238-s004.pdf]

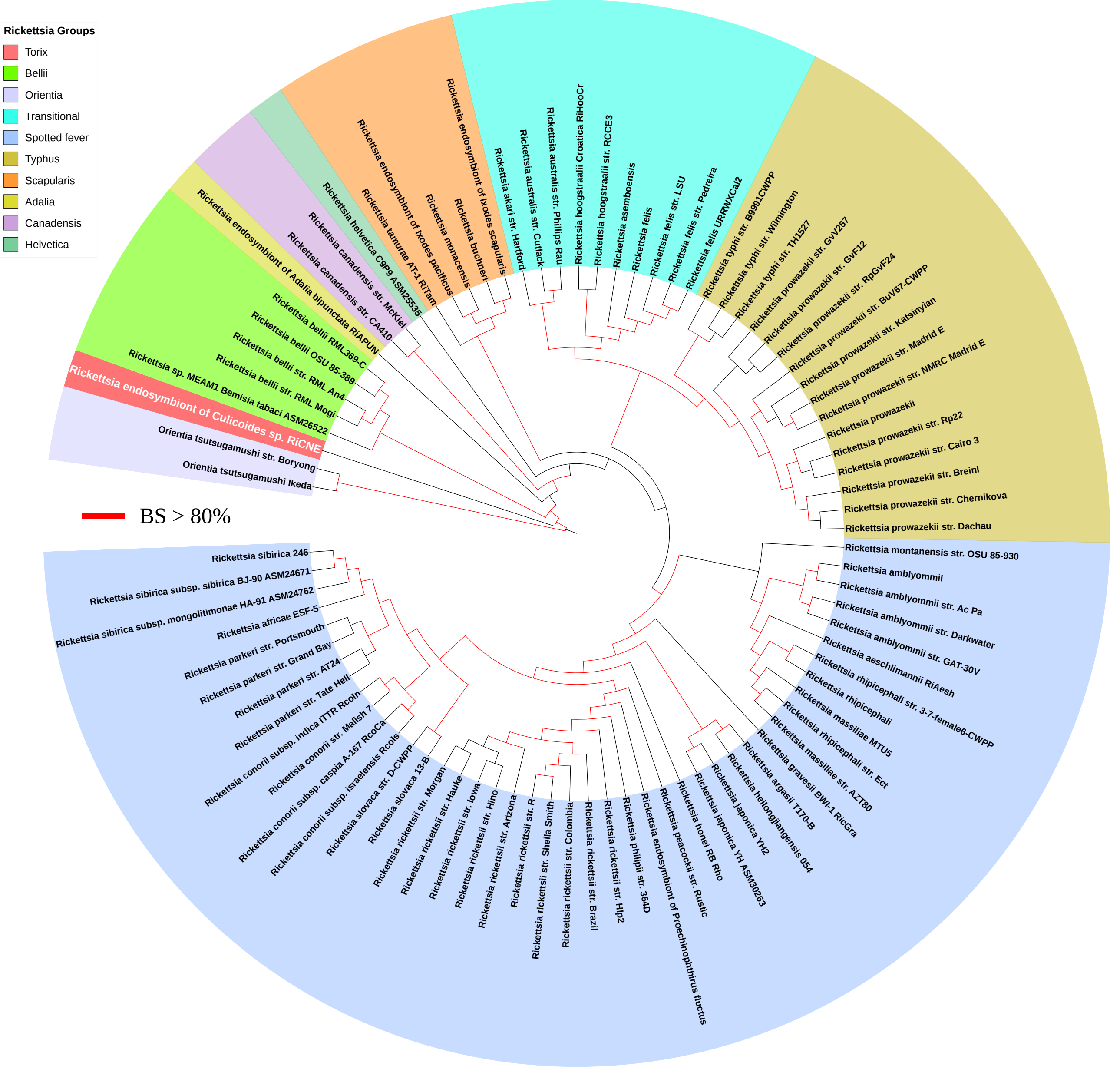

Figure S4. Cladogram of the complete core-genome phylogeny. Phylogenomic placement of the Rickettsia endosymbiont of C. newsteadii were inferred using maximum likelihood (RaxML, model: LG+G+I) from the concatenated protein allignments of 189 single copy ortholog genes. Support values are based on 100 rapid bootsrap replicates.
